# A Comprehensive Insight on the Health Benefits and Phytoconstituents of *Camellia sinensis* and Recent Approaches for Its Quality Control

**DOI:** 10.3390/antiox8100455

**Published:** 2019-10-06

**Authors:** Maram M. Aboulwafa, Fadia S. Youssef, Haidy A. Gad, Ahmed E. Altyar, Mohamed M. Al-Azizi, Mohamed L. Ashour

**Affiliations:** 1 Department of Pharmacognosy, Faculty of Pharmacy, Ain-Shams University, Cairo 11566, Egypt; maram.aboulwafa@pharma.asu.edu.eg (M.M.A.); fadiayoussef@pharma.asu.edu.eg (F.S.Y.); haidygad@pharma.asu.edu.eg (H.A.G.); prof_dean_zi@hotmail.com (M.M.A.-A.); 2 Department of Pharmacy Practice, Faculty of Pharmacy, King Abdulaziz University, P.O. Box 80260, Jeddah 21589, Saudi Arabia; aealtyar@kau.edu.sa; 3 Department of Pharmaceutical Sciences, Pharmacy Program, Batterjee Medical College, P.O. Box 6231, Jeddah 21442, Saudi Arabia

**Keywords:** biological activity, *Camellia sinensis*, phytochemistry, quality control

## Abstract

Tea, *Camellia sinensis*, which belongs to the family Theaceae, is a shrub or evergreen tree up to 16 m in height. Green tea is very popular because of its marked health benefits comprising its anticancer, anti-oxidant, and antimicrobial activities, as well as its effectiveness in reducing body weight. Additionally, it was recognized by Chinese people as an effective traditional drink required for the prophylaxis against many health ailments. This is due to the complex chemical composition of green tea, which comprises different classes of chemical compounds, such as polyphenols, alkaloids, proteins, minerals, vitamins, amino acids, and others. The beneficial health effects of green tea ultimately led to its great consumption and increase its liability to be adulterated by either low-quality or non-green tea products with concomitant decrease in activity. Thus, in this review, green tea was selected to highlight its health benefits and phytoconstituents, as well as recent approaches for its quality-control monitoring that guarantee its incorporation in many pharmaceutical industries. More research is needed to find out other more biological activities, active constituents, and other simple and cheap techniques for its quality assurance that ascertain the prevention of its adulteration.

## 1. Introduction

Natural products were widely employed for many centuries in home remedies, as well as in over-the-counter drug products and as raw materials for the manufacturing of various pharmaceuticals, cosmeceuticals, and nutraceuticals, both in developed as well as developing countries [1,2]. They provide a cost-effective natural solution for many ailments and possess nearly the same effectiveness as synthetic pharmaceuticals. They act via a multi-systemic effect but with relatively lower hazardous adverse effects in comparison to synthetic ones [3,4].

However, natural products are facing many challenges concerning their usage, exemplified by the difficulties related to the acquisition of sufficient pure amounts, particularly for those plants that grow in small quantities or at distant locations. Besides, a lot of problems are sparked regarding the standardization, as well as the determination, of a specific dose in a manner appropriate to the intended use. It is worthy to highlight that although natural remedies are relatively safe [5,6], the majority of lethal events reported in relation to herbal products’ consumption are mainly attributable to their poor quality that eventually hindered their usage as raw materials in many pharmaceutical industries [7].

Establishing internationally recognized guidelines for assessing the quality of natural products appeared to be mandatory. Sensory, macroscopic, and microscopic examinations are the first essential steps for establishing the identity and the degree of purity of herbal materials in regard to authentic/genuine samples of the material in question. Many analytical tools are employed in quality control, including both spectroscopic and chromatographic methods [8].

Tea, *Camellia sinensis* (L.) Kuntze or *Thea sinensis* F. Theaceae, is a shrub or evergreen tree up to 16 m tall [9]. Its leaves are alternate, exstipulate, lanceolate to obovate, up to 30 cm long, 2–5 cm broad, pubescent, sometimes becoming glabrous, serrate, acute or acuminate. Fresh leaves are often picked from late March and early April to July every year [10]. Green tea represents about 20% of the dried tea which is manufactured annually and is mainly consumed in Asian countries, such as Japan, owing to its relative safety and cheaper price in comparison to other beverages [11]. This was concomitantly reflected by the annual increase in its production, which is estimated to be 6.4 percent. It is noteworthy to mention that China, India, and Sri Lanka are the biggest tea-producing countries, respectively, as reported by the 22nd Tea session held by FAO-Intergovernmental Group. Kenya and Sri Lanka constitute the two major tea-exporting countries, respectively.

The chemical composition of green tea is highly complex, owing to its possessing different classes of chemical compounds, including polyphenols, alkaloids, proteins, minerals, vitamins, and amino acids. Besides, it is greatly popular because of its marked health benefits comprising its anticancer [12], anti-oxidant [13], anti-hypercholesterolemic [14], and antimicrobial activities [15], in addition to its effectiveness in reducing body weight [16]. Herein, due to the beneficial health effects of green tea that ultimately lead to its great consumption, this paper highlights its health benefits and phytoconstituents, as well as the recent approaches for its quality-control monitoring that guarantee its incorporation in many pharmaceutical industries. 

## 2. Health Benefits and Biological Activities of Green Tea (*C. sinensis*)

Green tea is well-known for its marked health benefits, and, additionally, it was recognized by Chinese people as an effective traditional drink required for the prophylaxis against many health ailments [17]. Most of its crucial health benefits and biological activities are discussed below and summarized in the supplementary data Table S1.

### 2.1. Antioxidant and Hepatoprotective Activity

In traditional medicines, green tea is considered a reference drug for many plant species with respect to its anti-oxidant potency [18]. This activity is mainly attributed to its polyphenols that are represented by its flavanols, which can be readily oxidized to the corresponding *o*-quinones, and thus function as hydrogen acceptors, as well as hydrogen donors [19]. The interaction with reactive oxygen species could be accomplished by the polyphenolic compounds existing in green tea via possessing different degrees of free radical scavenging properties, particularly toward oxygen-free radicals [20] and to some extent toward nitrogen (NO) species’ production inhibition [21]. The linkage of flavan-3-ol to gallic acid (GA) and the *O*-trihydroxy structure in the B ring are important criteria for the O_2_^−^ and NO scavenging activity of tea polyphenol [22]. Besides, green tea polyphenols possessing *ortho*-dihydroxyl functional groups, exemplified by epi-catechin and epi-catechin gallate, are good antioxidants that act in synergism with endogenous α-tocopherol [23].

The mechanistic potency for the anti-oxidant activity of green tea polyphenols (GTPs) was shown in different studies. GTPs act in different ways to suppress the active oxygen species generated from phenolic metabolites of benzene [24] and protect some treated oils from oxidation [25]. Other examples include increasing the excretion of carcinogenic products formed endogenously [26] and causing a marked enhancement in the salivary antioxidants in smokers, in both the short- and long term [27].

Green tea can effectively inhibit induced dose-dependent oxidative DNA damage and cell proliferation in the liver, as well as hepatotoxicity [28]. It induces the activity of some hepatic phase II enzymes, such as hepatic glutathione S-transferase (GST) [29], correcting the imbalances of the anti-oxidative system [30,31] and counteracting the induced lowered glutathione [32] in the liver. Epi-gallo catechin gallate (EGCG) and (−)-epigallocatechin-3-(3”-Omethyl) gallate are potent hepatoprotective agents, as they suppress cytotoxin-induced cell death, such as suppression of induced morphological change and induced cell death in a dose-dependent manner [33,34]. 

Additionally, high amounts of total phenolic compounds and caffeine prevents fat storage in the liver in high-fat diet cases [35]. Green tea is a source of highly important hexogen antioxidants that cannot be synthesized by our body and counterattack free radicals produced during metabolism of xenobiotics in the liver [36].

Moreover, green tea leaves can cause a significant reduction in elevated serum alanine aminotransferase (ALT), aspartate aminotransferase (AST), hepatic protein carbonyls, and ROS, and thus prevent the induced liver damage [13]. Dietary green tea extract treatment reduces liver injury during nonalcoholic steatohepatitis by decreasing the pro-inflammatory signaling [37]. In addition, the extract has a substantial therapeutic effect against alcohol-induced hepatic mitochondrial DNA damage [38]. 

### 2.2. Anticancer and Anti-Mutagenic Activity

Green tea polyphenolics GTPs are considered to be dietary chemopreventive compounds due to their potent effect on apoptosis and cell cycle progression inhibition [39]. It acts through different mechanisms to promote the function of P-glycoprotein-mediated efflux, reducing the cellular exposure to xenobiotics [40], and through the induction of the microsomal detoxification enzyme [41]. Green tea behaves as a potent anticancer, owing to its polyphenolics, which are effective against various chemically induced carcinogens, in addition to their pronounced effect on tumor suppression [42]. 

Many studies showed that green tea catechins inhibit matrix metalloproteinases (MMPs), which play an important role in tumor invasion and metastasis [43]. Epi-gallo catechin gallate (EGCG) is effective against cancer [44] via the prohibition of cell cycle progression [45]. It inhibits fatty-acid synthase (FAS) [46], modulates protease activity during endothelial morphogenesis [47], and inhibits the vascular endothelial growth factor (VEGF) family [12], so it prohibits angiogenesis, which is a crucial step in the growth and metastasis of cancers [48]. Additionally, the cytotoxic activity of EGCG is also due to its auto-oxidation products, which also possess equivalent cytotoxic activities to EGCG [49]. Moreover, methylxanthines and polysaccharide inhibit tumor metastasis [50]. 

Green tea exerts a significant anticarcinogenic effect against many different types of cancer. Oral or topical application of green tea polyphenols possesses a considerable potential to antagonize the tumorigenic effects of some polycyclic aromatic hydrocarbons on skin [51]. Regarding photo-carcinogenesis, oral administration of green tea inhibits the formation of skin lesions produced by UVB light in a dose-dependent manner and prolongs the time of tumor appearance and decreases their number [52]. However, topical application of GTPs or EGCG protects against UVB-induced local and systemic immune suppression and skin cancer induction [53]. Also, it was found that green tea consumption reduces UVB-induced skin redness and causes significantly fewer UVA+B light-induced skin papillomas and tumors, and thus diminishes the incidence of skin cancer [54]. Moreover, many studies reported that green tea can be used in the treatment of malignant melanoma [55]. Green tea treatment was also found to decrease the induced fore-stomach, duodenal, large intestine, and colon tumors, and it can protect against the metastatic process in gastric cancer cells and strongly inhibit the growth of hepatocellular carcinoma cells [56,57,58]. Furthermore, green tea exerted an antagonistic effect on induced micronuclei and apoptosis in colonic crypt cells [59]. Moreover, it inhibits the hyperoxidation of membrane phospholipids, which reflect the degree of DNA damage and carcinogenic alteration [60], and it causes less metachronous colorectal adenomas incidences in patients who have undergone the complete removal of colorectal adenomas [61].

Regarding lung cancer, green tea infusion revealed a notable efficacy in the alleviation of lung cancer via EGCG, which reduces the mean number of lung tumors, causes a decline in the multiplicity of lung adenomas, and counteracts metastasis with concomitant inhibition of induced lung tumorigenesis, acting synergistically with other cancer preventive agents [62,63]. Furthermore, EGCG also inhibits lung cancer stem cells [64] and stimulates apoptotic induction in human lung cancer cells [65].

Concerning leukemia, crude catechins exemplified by (+)-gallocatechin, EGC, EC, EGCG, and ECG have a suppressive effect on erythroblastic leukemia cells in a dose-dependent manner [66]. EGCG inhibits the growth of human leukemic cell lines without causing side effects and in a specific way [67]. Furthermore, EGCG exhibits antileukemic activity by inducing the differentiation of the leukemia cells, suppressing their proliferation [68] and inducing apoptosis of B-cell and T-cell chronic lymphocytic leukemia in a dose-dependent manner, without affecting healthy B-and T-cells [69]. In addition, green tea intake has a small protective effect against acute myeloid leukemia [70]. Moreover, breast cancer could be ameliorated via the daily consumption of green tea catechins. It could effectively reduce mortality triggered from induced mammary carcinogenesis, in addition to reducing the average sizes of tumors [71]. Polyphenon E, which contains about 58.4% EGCG, was noticed to exert a mild inhibitory effect on the early promotion stage [72]; however, catechins may act in the post-initiation stage, but not in a dose-dependent manner, in addition to reducing recurrences [73,74]. Recently, it was found that green tea drinkers have no excess risk for epithelial ovarian cancer [75]. Furthermore, green tea has proved to have antiproliferative effects against human prostate cancer cells [76] and can lead to cell growth inhibition in some prostate cancer cells [77] showing potent inhibition of 5α-reductase enzyme, which may be involved in the development of benign prostatic hyperplasia, hirsutism, and prostate cancer [78,79]. GTPs also strongly inhibit the growth of renal cell carcinoma cell lines in a concentration-dependent manner [80]. It is worthy to highlight that green tea catechins possessing the gallate group act as anticancer agents on glioblastoma cells and can prevent the inflammatory processes associated with aggressive glioma tumor growth [81,82]. Nowadays, green tea serves as a diet-derived immune-modulatory chemopreventive agent; as many of its contained flavonoids stimulate natural killer (NK) cell activity, so it may be used in the treatment and prevention of malignant diseases [83]. 

Meanwhile, aqueous extract of green tea has a marked antimutagenic activity against different major classes of dietary and occupational carcinogens [84]. This activity may be attributed to different mechanisms, such as improving the fidelity of DNA replication [85], affecting carcinogen metabolism [86], direct interaction between the reactive genotoxic species of the various promutagens, and nucleophilic tea components. In addition, it causes a potent inhibition of the cytochrome P450-dependent bioactivation of the promutagens [87], acting intracellularly as a blocking or suppressive agent [88], and potent dose-dependent antigenotoxic activity [89]. Besides the antimutagenic activity of green tea, it has anticlastogenic effects [90].

### 2.3. Antimicrobial and Antiviral Activity 

Many studies reported the antimicrobial activity of green tea. Green tea is effective against *Staphylococcus epidermidis*, *Staphylococcus aureus*, and *Vibrio cholerae O1*, owing to the bactericidal catechins that primarily cause defection in the bacterial membranes [91]. Green tea is also effective against various bacteria that cause dental caries, such as *Escherichia coli*, *Streptococcus salivarius,* and *Streptococcus mutans* [92]. EGCG and gallocatechin gallate (GCG) markedly inhibit the secretion of extracellular Vero toxins from enterohemorrhagic *Escherichia coli* cells into the culture supernatant fluid [93]. Raw extract of green tea, especially gallocatechin gallate (GCG), is able to suppress 1-deoxy-d-xylulose 5-phosphate reductoisomerase activity, which is an antimicrobial target [94]. Furthermore, it can also reduce the lethality of ricin toxin [95], but it has a poor activity against *Babesia divergens* that infect cattle [96]. 

EGCG can inhibit the activity of *Salmonella* *typhimurium* type III, and thus reduce the bacterial invasion into host cells [97]. Green tea extract is bactericidal against Gram positive bacteria and bacteriostatic against Gram negative ones, with less antifungal activity against *Aspergillus niger* and *Penicillium chrysogenum* [98]. 

In addition, tea polyphenols have the ability to inhibit the development and growth of bacterial spores, as in case of *Bacillus stearothermophilus* and *Clostridium thermoaceticum*, due to their ability to decrease the heat resistance of these bacterial spores when added at high temperature [99]. However, chlorogenic acid induces the apoptotic markers through excessive potassium efflux and an apoptotic volume reduction, which induces cytosolic calcium uptake and cell cycle arrest in *Candida albicans*, in addition to its ability to induce caspase activation and DNA fragmentation [100]. 

Tea exhibits antiviral activity against human viruses and serves as a diet-derived immune-modulatory chemopreventive agent, as many of its contained flavonoids stimulate NK cell activity, so it may be used in the treatment and prevention of viral diseases [83]. Recently, it was found that EGCG is capable of inhibiting the Brazilian strain of Zika virus entry [101]. Additionally, topical application of EGCG can be used to prevent the sexual transmission of HIV, as it disaggregates existing amyloid fibrils termed semen-derived enhancers of viral infection fibers and inhibits the formation of new ones [102]. 

### 2.4. Anti-Schisosomiasis and Antiparasitic Activity

Green tea could be able to protect liver cells in mice after being infected with *Schistosoma mansoni*, and thus decreases cellular necrosis and regenerates total protein and glycogen levels partially. This could be achieved through suppression of the oxidative stress, owing to its scavenging properties toward free radicles [103]. Moreover, green tea has an antiparasitic activity as it noncompetitively inhibits the activity of *Toxocara canis* [104].

### 2.5. Cardioprotective Activity

Green tea could improve the risk factors for heart disease [105], as it significantly reduces total cholesterol, low density lipoprotein (LDL) cholesterol, and blood pressure [106]. It also improves microvascular function and skin oxygen tension in both older and younger populations [107]. Nonfermented Chinese green tea is considered to be an ideal beverage to prevent the incidence of coronary heart disease. Its consumption, together with its catechin-rich fractions, lowers the risk of coronary heart diseases through delaying atherogenesis by significantly preventing endothelial cell induced LDL oxidation and foam cell formation [108]. The inhibition of advanced glycation end products formation in collagen represents another important mechanism for the protective effects of green tea catechins against cardiovascular diseases [109].

The consumption of green tea decreases the risk of myocardial infarction (MI) in a dose-dependent manner up to ≥4 cups/day [110], as it reduces cardiac hypertrophy, improves systolic and diastolic dysfunction, restores the antioxidant enzyme activity, and stimulates the glucose pathway and mitochondrial function with reduced apoptosis after MI [111]. High dietary intake of green tea may be useful in the reduction and prevention of cardiac injury following ischemia [112]. 

The flavonoids that exist in green tea perform its cardio-protective effect by improving the reserve in coronary flow velocity [113]. EGCG is able to reduce both arsenic- and doxorubicin-induced cardiotoxicity [114]. It reduces the inflammation and preserves the cardiac function, with lowered mortality rate [115]. Concerning EC, it may be involved in treating cardiac arrhythmia [116]. Meanwhile, EC supplementation has a cardioprotective effect without changing blood pressure, arterial stiffness, or the blood lipid profile [117].

Additionally, green tea can be used as anti-hypercholesterolemic agent by acting in several ways, such as enhancing hepatic excretion of cholesterol or inhibiting absorption of cholesterol in the alimentary tract [118]. This is accompanied by increasing fecal bile acids and cholesterol excretion, resulting in the lowering of the plasma cholesterol [119] and the lowering of LDL oxidation by increasing cellular antioxidant status or inhibiting oxidizing enzyme activities in the arterial wall [14]. Other possible mechanisms include diminishing the levels of α-ketoglutarate and pyruvate dehydrogenases enzymes, which are vital in the cholesterol biosynthesis [120] or inhibition of the rate limiting enzyme of cholesterol biogenesis, squalene epoxidase (SE) [121]. Green tea also causes a prolongation in LDL oxidation lag time by flavonoids [122], inhibition of serum triglyceride elevation [123], and prevention of fat storage in the liver, lowering blood lipids and increasing fecal excretion of triglycerides [35]. 

Fresh green tea fermented under nitrogen gas produces γ-aminobutyric acid (GABA)-rich tea, which was proved to prevent the occurrence of hypertension [124]. Theanine exerted a significant decline in blood pressure following a dose-dependent manner [125]. EGCG and EGC were found to inhibit dopa decarboxylase enzymes in a concentration- and time-dependent manner, which is a known target for drugs used in hypertension [126]. EGCG that was chronically infused in the hypothalamic paraventricular nucleus (PVN) attenuates hypertension by chronic inhibition of ROS, in addition to regulating the balance of neurotransmitters, as well as cytokines, in the PVN [127]. Green tea extract was found to prevent high angiotensin II dose-induced hypertension and the accompanied organ damage by preventing or scavenging superoxide anion generation [128]. Decaffeinated green tea extract also reduces the metabolic syndrome through reduction of the formation of ROS, which results in lowered blood pressure [129]. 

Regarding green tea anti-thrombotic activity, it was found that unprocessed tea extracts can significantly reduce the levels of thromboxane-B2 and then eliminate the aggregation of platelets to produce microthrombi, while processed ones are unable to form any inhibition, significantly owing to the presence of a heat-labile compound [120]. Possibly, green tea has a fibrinolytic effect [105]. In addition, the catechins inhibit induced platelet aggregation in vitro in a dose-dependent manner, without changing the coagulation parameters [130]. The tea can also be used to prevent red blood cell hemolysis [131]. Different green tea extracts have different degrees of inhibition of dehydration of stored sickle cells, and this inhibitory activity increases by increasing the number of hydroxyl groups [132]. 

### 2.6. Antidiabetic and Anti-Obesity Activity

Green tea is used for the amelioration of diabetes and its complications, acting as a renoprotective in diabetes mellitus, in addition to the ability of EGCG to inhibit angiogenesis that is involved in many diseases, such as diabetic retinopathy [48,133]. Furthermore, EGCG can help in controlling hyperglycemia and alleviating diabetes complications via reducing plasma glucose, insulin level, and liver and kidney weight [134]. Recently, it was found that EGCG is useful for resensitizing insulin-resistant muscle [135]. 

The administration of GTPs significantly increases glucose tolerance and reduces induced elevated serum glucose levels [136]. Floratheasaponins A, B, and C in the flower buds of green tea exhibit potent inhibitory effects against sucrose-induced serum glucose elevation [137]. Green tea water-soluble polysaccharide fraction has an inhibitory effect against α-amylase, which leads to decreased blood glucose levels [138], along with galloyl moiety in catechin, which increases the inhibition of pancreatic α-amylase, due to enhanced association with the enzyme active site [139]. 

Regarding obesity, green tea extract is a well-tolerated natural product for the management of obesity by reducing fat digestion through marked inhibition of digestive lipases, such as gastric and pancreatic lipases, especially by saponins, which lead to altered lipid emulsification in gastric or duodenal media [140]. In addition, it stimulates thermogenesis/energy expenditure and fat oxidation mainly by EGCG and caffeine [16], without significant differences in either plasma cholesterol or blood pressure [141]. Furthermore, chronic usage of short-time decoction of green tea decreases perirenal and epididymal adipose tissues and weight gains in high-fat diet cases [35]. 

Recent studies confirm that a mixture of green tea catechins and caffeine has a beneficial effect on body-weight management through sustained energy expenditure, fat oxidation, and preservation of fat-free body mass [142]. Polyphenols such as EGCG can increase lipolysis, in addition to possessing anti-adipogenic effects through being a fatty-acid synthase (FAS), which is a possible therapeutic target for appetite and weight control [143,144]. Polyphenols have “exercise mimetic” properties through exercise-inducible pathways [16]. 

### 2.7. Gastrointestinal Tract Problems Relieving Activity 

The purified green tea EC can cause both endothelium-dependent and -independent mesenteric arteries relaxation [145]. However, EGCG preserves the aortic thickness and regenerates elastin content, due to its anti-inflammatory effect, so could prevent abdominal aortic aneurysm [146]. Furthermore, high green tea consumption is associated with a reduction in the risk of precancerous chronic atrophic gastritis [147].

In addition, floratheasaponins A, B, and C in flower buds exhibit potent inhibitory effects against ethanol- and indomethacin-induced gastric mucosal lesions, so green tea seems to have a gastro-protective effect [137]. However, daily intake of green tea is able to alter the growth and composition of the intestinal flora and modulate the genesis of potentially harmful agents like *Clostridium difficile* and *Clostridium perfringens*, due to the effect of its components, such as vitamin C and polyphenols [148]. Green tea has recently proved to modulate the fecal microbiome and thus endogenous metabolites [149]. 

### 2.8. Neuroprotective Activity

Green tea extract has a protective effect on the ischemia/reperfusion-induced brain injury and behavior deficit. It also reduces the number of ischemia/reperfusion-induced apoptotic neuronal cells [150]. GTPs, EGCG, ECG, EGC, and EC are able to protect synaptosomes from induced lipid peroxidation damage [151]. EGCG alone has a protective effect against stress-induced neural injuries [152]. EGCG was shown to be easily absorbed from the digestive tract and penetrate the brain, reaching levels similar to those found in lung, liver, kidney, and others [153]. It has a neuroprotective effect against neuronal damage following transient global ischemia in the gerbils acting by different mechanisms as angiogenesis in the early stage of ischemic stroke promoting [154,155]. Regarding neurotoxicity, l-theanine has a protective effect against cadmium-induced neurotoxicity by reducing brain cadmium levels and oxidative damage, which lead to neurodegenerative diseases [156].

In addition, GTPs can be considered therapeutic agents to alter brain aging processes by serving as neuroprotective agents in major neurodegenerative disorders, such as Parkinson’s disease and Alzheimer’s disease [157,158]. In addition, green tea catechin intake may be useful in the improvement of the morphologic and functional changes that occur naturally in the accelerated senile brains [159]. EGCG and EGC were found to be inhibitors of dopa decarboxylase enzyme in a concentration- and time-dependent manner [126]. Green tea containing high levels of EGCG prevents the loss of tyrosine hydroxylase (TH)-positive cells in the substantia nigra [160]. Both tea and EGCG, when used alone or with Parkinson’s disease’s inducers, can decrease the neuronal nitric oxide synthase (nNOS) expressions in the substantia nigra, which provides a neuroprotective effect [161].

Regarding Alzheimer’s disease, EGCG may be beneficial for its prevention, as it has protective effects against amyloid ß-induced hippocampal neuronal apoptosis [162] in which neuronal loss is accompanied by the deposition of amyloid ß protein in senile plaques [163]. EGCG regulates the growth and survival of astrocytes without being cytotoxic. Green tea catechins possess an inhibitory effect against *β*-secretase, which is known as one of the most important amyloid precursor protein cleaving enzymes in Alzheimer’s disease [164]. EC reduces amyloid-β-induced *β*-secretase-1 expression and thus inhibits the most toxic amyloid-β aggregates [165]. 

The theanine existing in green tea enhances the memory and learning ability, owing to its significant effect on the release or reduction of memory- and learning-linked neurotransmitters, such as dopamine and serotonin [125,166]. Polyphenols also have a potential role in the prevention/treatment of dementia [167]. It was proved that combined ingestion of EGC and GA increases the learning ability in a better way than EGCG, which reaches the brain parenchyma at a very low concentration [168]. Additionally, theanine may affect emotions by interacting with neurotransmitters in the brain, due to a modulation of synaptic transmission [42]. Moreover, drinking theanine-rich and low-caffeine green tea has anti-stress effects, as theanine, EGC, and arginine together can counteract the effect of caffeine and EGCG on psychosocial stress-induced adrenal hypertrophy [169]. Theanine also produces a marked relaxation effect due to its rapid absorption and transportation to the brain in 30 min, without suffering any metabolic degradation [125].

### 2.9. Anti-Inflammatory, Analgesic, Antipyretic, and Anti-Allergic Activity

Green tea is considered to be a potent anti-inflammatory and antipyretic agent [170,171]. EGCG reduces nitric oxide production via its gallate structure [172]. It inhibits the endothelial cell growth which leads to the inhibition of angiogenesis that is involved in many diseases, such as chronic inflammation [48]. The non-polyphenolic fraction of green tea represented by Pheophytin a and b exerts suppressive activities against the activation of human polymorphonuclear neutrophils (PMNs) associated with inflammatory reactions in a dose-dependent manner [173]. Moreover, green tea extract/tablets showed a high efficacy in controlling pain, particularly in patients suffering from osteoarthritis as well as [174]. 

Regarding the anti-allergic activity of green tea, polyphenols are considered to be the major inhibitory components of hot-water-soluble extracts of green tea against histamine release from mast cells [175]. In addition, green tea has an anti-allergic effect due to leaf saponins [176] and floratheasaponins from flower buds that possess anti-allergic activities, as well [177]. It was reported that methylated EGCG blocks the production of two compounds in the body mainly involved in producing an allergic response, histamine and immunoglobulin E (IgE), and works by triggering and sustaining allergic reactions. It is worthy to mention that green tea can activate heat-generating mechanisms in the body such that a fall in core body temperature can be lowered upon cold exposure [178].

### 2.10. Skeletomuscular System Relieving Activity

EGCG has direct vasodilator action in skeletal muscle and increasing muscle microvascular blood flow [179]. It can induce myogenic differentiation [180] and increase the recovery of muscle mass and function [181]. Moreover, EGCG induces apoptotic cell death of osteoclasts in a dose-dependent manner with no effect on osteoblasts [182]. Appropriate concentrations of EGCG have an anti-inflammatory effect in the treatment of collagen membranes and thus can be used in bone regeneration [183]. It was also found that green tea has a protective effect against induced bone and hyaline cartilage alteration [184]. Furthermore, EGCG enhances osteoprotegerin synthesis, which is secreted from osteoblasts, and suppresses osteoclastic bone resorption, especially in the elderly [185]. Meanwhile, EGCG has the potential to inhibit the development of arthritis, as pretreatment with EGCG inhibits the release of induced lactate dehydrogenase in human chondrocytes of osteoarthritis cartilage [186]. Green tea extract can also be used as an adjunctive treatment, because it can control the pain and improve the knee-joint physical function in adults with osteoarthritis [187]. 

Green tea can be useful as a medicament for treatment of infected root canals, as Japanese green tea extracts have antibacterial and bactericidal effects against some facultative anaerobes and obligative anaerobes [188]. It also has a high amount of fluoride, which may help strengthen teeth and bones and reduce tooth decay [189]. Besides fluoride, green tea has a high concentration of polysaccharides, such as pectin, which enhances the inhibition activity against *Streptococcus mutans*, thus preventing dental caries [190]. Green tea is also effective against other various bacteria that cause dental caries, such as *Escherichia coli* and *Streptococcus salivarius* [92]. The combination of green tea and miswak (*Salvadora persica* L.) extracts exhibits synergistic antiplaque properties [191]. Additionally, green tea can be used to prevent periodontal diseases, due to its matrix metalloproteinases (MMPs) inhibitory activity in a dose-dependent manner [192], with particular reduction in their secretion in gingival fibroblasts [193]. 

### 2.11. Miscellaneous Activity

Moreover, green tea experienced a lot of pronounced activities in relieving skin damage and promoting wound healing. Oral or topical treatment of GTPs prevents damages such as ultraviolet (UV)-induced sunburn response, immuno-suppression, and photo-aging [194], such as dermal extracellular damage [195] and DNA damage [196]. Treating the skin with green tea extracts inhibits induced erythema response in a dose-dependent manner, especially when treated with EGCG and ECG [197], due to the ability of EGCG to inhibit UVB-induced oxidative stress [198]. In addition, green tea ethanol extract is effective in the healing process of surgical wounds, as it decreases the healing duration [199]. Recently, it was found that tannase-converted green tea extract can be used in cosmetics as a skin anti-wrinkling or depigmenting agent [200], as tannase-derived bioconversion, which occurs on catechins compositions in green tea, was found to be useful in improving the antioxidant and radicals scavenging activities [201]. 

It is notable that anti-amyloidogenic activity has been attributed to oxidized EGCG comparable to the intact molecule, as it has a disruptive effect on preformed fibrils more than the native form [202]. Green tea revealed a high potency in the prohibition of proMMP-9 and MMP-9 activities, which play an important role in the development of pulmonary hypertension [203]; meanwhile, l-theanine proved efficacy in the alleviation of oxidative stress-induced airway inflammation in asthma [204]. Recent studies showed that dietary supplementation enriched with green tea promotes the antioxidant defense system in plasma and thus provides protection against oxidative damage induced by both short-term muscular endurance test and long-term strength training [205]. Additionally, the tea extract can prolong the time to exhaustion [206] and improve vascular function and physical performance in healthy individuals [207].

Green tea extract has a protective effect against induced reproductive toxicities and reduced testicular androgen receptors [208]. In addition, green tea improves induced damage of the reproductive system; this is probably due to its high catechins content [209], as catechins have a quenching effect on the reactive oxygen species that lead to oxidative stress in male and female reproduction systems and cause infertility [210]. EGCG increases the total efficiency of fertilization through improving the in vitro penetration rate of the sperms [211]. Meanwhile, oral administration of polyphenone-60 (P-60), green tea extract catechins, can stimulate goiter and reduce weights of the body, testis, and prostate gland, along with elevation in plasma thyroid stimulating hormone (TSH), luteinizing hormone (LH), and testosterone levels and reduction in tri-iodothyronine (T3) and thyroxine (T4). P-60, as a whole, and some of its constituents, can inhibit human placental aromatase activity which then can be used as preventive agents against benign prostatic hypertrophy (BPH) [212].

Regarding the effect of green tea extract on eyes, it has potential cataracto-static ability and can delay the progression of lens opacity, as it decreases smoking-induced frequencies of micronuclei in peripheral-blood lymphocytes [213]. It also counteracts the oxidative insult caused by cigarette smoke that causes oxidative damage to the constituent molecules and consequent lenticular opacity [214]. GTPs can bind to the cleft between the domains of human γB-crystallin protein, which is then protected from UV induced-oxidative stress [215].

Green tea may have selective protective effects within the body, especially on the kidney [216]. Green tea tannins, especially EGCG and ECG, cause a dose-dependent decrease in the progression of renal failure [217]. Recently, it was found that green tea extract can prevent the induced lipid peroxidation and antioxidant depletion in the kidney [218].

## 3. Effect of Green Tea Administration on the Bioavailability of Other Drugs

Saponins from flower buds have a stimulating effect on gastrointestinal transit and prohibiting effects versus pancreatic lipase [219], while green tea polyphenols, EGCG, ECG, and catechin gallate (CG), inhibit the binding and efflux of drugs by P-glycoprotein [220], with EGCG being the most potent inhibitor, since it remains in the intestine, whereas EGC and EC are rapidly absorbed [221].

First, neither caffeine nor flavonoids were likely to be responsible for the increase in CYP4A1, as well as expression [222]. Then, it was found that flavanols are not responsible for the effects of tea on the cytochrome P450 system, but caffeine could be responsible for the increase in CYP1A2 [223]. Controversially, EGCG was found to be able to inhibit CYP1A transcription but in a less effective manner than whole green tea extracts [224]. It causes a decrease in the level of intestinal bacteria of *Clostridium* species, which leads to a decrease in the drug-metabolizing enzyme cytochrome P450 3A (CYP3A) expression level and activity [225]. Recently, it was found that oligomerized EGCG can improve pharmacokinetic parameters in oral drug delivery systems [226].

## 4. Phytoconstituents of Green Tea (*C. sinensis*)

Green tea’s chemical composition is highly complex, owing to its richness with different classes of chemical compounds, which are described below.

### 4.1. Polyphenols

These represent the most important group of green tea leaf components, and thus they are regarded as the popular dietary source of polyphenols that accounts for 50–70% of tea water extract [227]. Basically, these polyphenols are particularly flavonoids that are considered biosynthetically produced in considerable amounts ranging from 0.5% to 1.5%, with more than 4000 varieties [228]. The major abundant flavonoids in green tea comprises catechins (flavan-3-ols), which represent about 30–40% of its dry weight [229]. Four major catechins predominate in green tea: (−)-epigallocatechin-3-gallate (EGCG) **(1)**, epigallocatechin (EGC) **(2)**, epicatechin-3-gallate (ECG) **(3)**, and epicatechin (EC) **(4)**. The former accounts for 59% of the total catechins and about 10% of dry weight [230]. However, the three latter catechins represent approximately 19%, 13.6%, and 6.4% of the total catechins, respectively [231]. These different structures are very important for their pharmacological usage [20]. 

Additionally, tannins are the second major polyphenols present in tea products, and they are responsible for the astringency of green tea [232]; that is why green tea is considered to be a selected food-grade plant extract which is also called ‘commercial tannins’ [233]. In addition, phenolic acids comprising caffeic acid **(5)**, chlorogenic acid **(6)**, coumaric acid **(7)**, gallic acid (GA) **(8)**, and its quinic acid ester **(9)**, as well as flavanols represented mainly by kaempferol **(10)**, myricetin **(11),** and quercetin **(12)**, are also found [234]. Additionally, proanthocyanidins like epiafzelechingallate-(4β→8)-epicatechingallate (EAG-4β→8-ECG) **(13),** epiafzelechingallate-(4β→6)-epicatechingallate (EAG-4β→6-ECG) **(14)** [235], epigallocatechin-(4β→8)-epigallocatechingallate (EGC-4β→8-EGCG) **(15),** and epigallocatechingallate-(4β→8)-epigallocatechingallate (EGCG-4β→8-EGCG) **(16)** [236], as well as and traces of flavones [237], like vitexin pigment **(17)** [238], are present.

### 4.2. Xanthine Bases/Purine Alkaloids

They are represented mainly by caffeine **(18),** which is considered to be the second major constituent of the dry leaf [239]; meanwhile, its two metabolites, theophylline **(19)** and theobromine **(20)**, are also present but in smaller amounts. 

### 4.3. Triterpenoid Saponins

They are represented by floratheasaponin A, B, C, D, E, and F **(21–26)** [137], which exist at high concentrations in seeds and flowers [240].

### 4.4. Amino Acids

Amino acids constitute about 1–4% of dry weight and are represented by arginine **(27)**, aspartic acid **(28)**, glutamic acid **(29)**, glutamine **(30)**, and serine **(31),** as well as theanine **(32)** or 5-*N*ethylglutamine, which account for more than 90% of the whole amino acids present in the leaves of *C. sinensis* [241]. It is worthy to mention that theanine is the major amino acid that exists in the largest amounts, comprising about 1–2% of the dry weight of the green tea leaf [242], and thus it is considered the third major constituent of dry leaf [239]. Furthermore, it is recognized to be the only amino acid that exists solely in tea plants to which the flavor, as well as the exotic taste of green tea, is attributed [125]. Tryptophan **(33)**, glycine **(34)**, tyrosine **(35)**, valine **(36)**, leucine **(37)**, threonine **(38),** and lysine **(39)** are also found. 

### 4.5. Minerals and Trace Elements 

They include Al, Ca, Cr, Co, Cu, F, Fe, K, Mg, Mo, Mn, Na, Ni, P, Se, Sr, and Zn, which constitute about 5% of dry weight [243]. 

### 4.6. Miscellaneous Compounds

These include proteins, which constitute about 15–20% of dry weight, whose enzymes represent an important fraction (e.g., carotenoid cleavage enzymes). Moreover, carbohydrates, such as pectin **(40)**, glucose **(41)**, fructose **(42)**, and sucrose **(43)**, represent about 5–7% of dry weight. Additionally, lipids are found and represented by linoleic and α-linolenic acids **(44 and 45)**, as well as sterols such as stigmasterol **(46**). Green tea is also considered a source of vitamins, such as vitamin B **(47)**, vitamin C **(48)**, and vitamin E **(49)**. Chlorophyll **(50)** and carotenoids **(51)** are important tea pigments that play a crucial role in enhancing the quality of green tea [244]. Moreover, volatiles, such as aldehydes, alcohols, esters, lactones, hydrocarbons, and terpenoids, are mainly responsible for the formation of tea aroma [245]. However, policosanol **(52)**, which is composed of a group of health-promoting bioactive long-chain aliphatic alcohols, is exceptionally rich in green tea [246]. 

**Figure 1:**
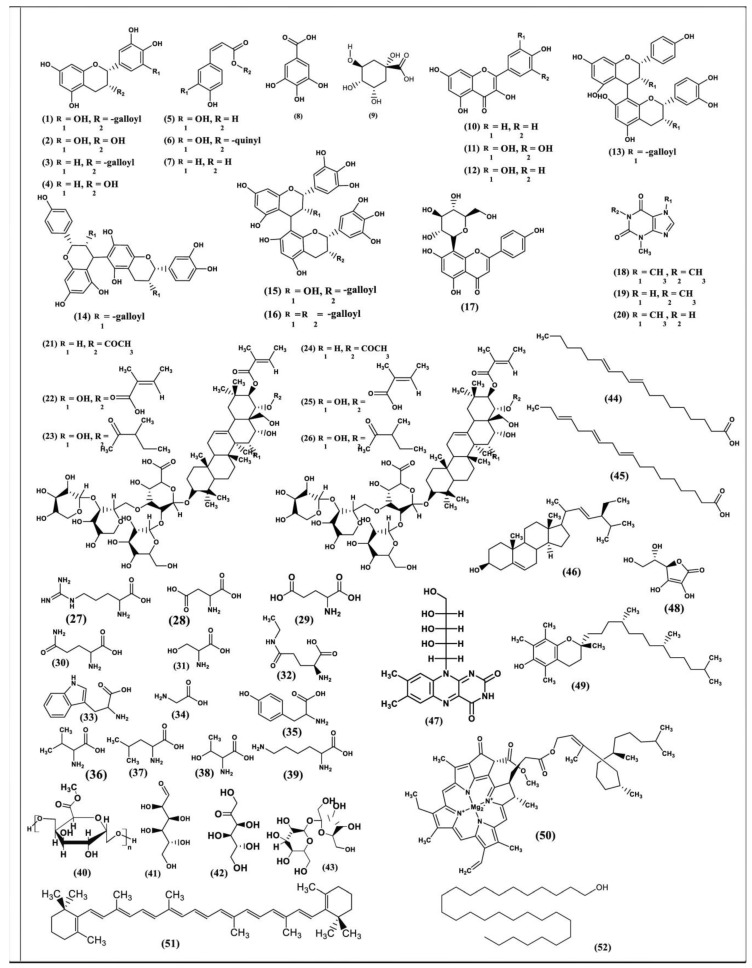
Most of the phytoconstituents isolated from green tea (*C. sinensis*).

Most of the phytoconstituents isolated from green tea (*C. sinensis*) are represented in Figure 1.

## 5. Recent Approaches for Monitoring the Quality of Green Tea (*C. sinensis*)

Many approaches were adopted for assessing the quality control of green tea, owing to the great consumption of green tea worldwide and the high variability in its quality that ultimately influences its biological activities, as well as its cost. In addition, green tea is highly susceptible to adulteration with various non-green-tea materials, and this necessitates its strict quality-control monitoring. The various techniques that were previously adopted for monitoring the quality of green tea are summarized below.

### 5.1. Morphological Aspects

Green tea processed from fresh leaves picked at an early stage is considered to possess a high quality that consequently reduces with time [10]. The amount of pubescence on leaf epidermis is an important morphological marker for the quality of green tea, as the tender tea was also found to have more pubescence than old one [32]. Tea with plenty of pubescence has a better taste depending on the profiling of tea leaf pubescence metabolites [247]. 

Moreover, tea leaves should not be stored for more than two months at room temperature, since they become oxidized. Bioactive-ingredients extraction from green tea through ultrahigh pressure extraction (UPE) technique is characterized by being less time-consuming, producing higher extraction yields, consuming less solvent, and possessing higher purity with minimal heat generation, which may lead to thermal degradation of bioactive components. Its micromechanism is the disruption of cellular organelle which enhances the diffusion and mass transfer [248].

### 5.2. Physical Quality Parameters 

#### 5.2.1. Aroma and Flavor 

The number of volatile constituents existing in green tea and the percentages in which they exist greatly influence its aroma and flavor, which are undoubtedly considered determinant factors when judging its quality [249,250]. Green tea aroma is severely affected by any unfavorable conditions that take place during manufacture or/and storage; thus, it should be monitored via objective, simple, easy, and rapid methods, like an odorant sensor [251]. Electronic nose (E-nose) is a fast, reliable, and robust technology which is easy-to-use for the evaluation of tea aroma and cost-efficient. It is able to handle continuous real-time controlling of odor at certain places in the field, over hours, days, weeks, or even months [252,253]. 

E-nose is widely used for qualitative purposes, such as distinguishing different types of tea, defining multiple brands, and classifying samples obtained by various processing methods [254]. It is noteworthy to mention that little research exists that describes the use of E-nose for evaluating the variable quality grades of green tea [253]. Chemometric tools make it possible to use the E-nose technique in the field of quality control of green tea, as it was found that combining the E-nose technique with the Support Vector Machine (SVM) classification tool can be used to develop a discrimination model for green tea’s quality and identifying tea grades [255]. In addition, E-nose, possessing an array of six metal oxide semiconductor sensors, was also used to obtain olfactive fingerprints of the volatile compounds in the infusions headspace. This is concomitantly associated with another chemometric tool such as Linear Discriminant Analysis (LDA) of the resulted data that can be used as a model for fast characterization of green teas subjected to long-term storage. Additionally, classifying unknown samples into aged and fresh samples was also made possible by the utilization of the previously described E-nose [256].

Regarding its flavor, it was found that rated acceptance of green tea is decreased as bitterness increases [257]. Taste of tea infusions is crucial for both judging tea quality, as well as acting as a reference in tea classification [258]. The taste quality of green tea infusions prepared using purified water is higher than those prepared using mineral water or tap water, while decreasing the pH value in the case of mineral water partially improves the taste quality [259]. 

Expert tasters who are well qualified do flavor-quality evaluations by using their own experience to describe multiple quality levels of tea infusions that are in some cases very tedious to be comprehended by consumers [10]. However, in most cases examining flavor manually using human tasting is not fully accurate, and sensitivity may be reduced by time, owing to prolonged exposure [249]. Besides, the results may be greatly altered by many factors, including emotional ones, feelings of exhaustion, and infection attacks [260]. Alternatively, electronic tongue (E-tongue), which is also termed artificial tongue or taste sensor, was used since the 2000s and is effectively capable of classifying different tea categories and evaluating their quality levels according to their content of amino acids, catechins, caffeine, and polyphenols [10,258]. 

E-tongue coupled with pattern recognition using Artificial Neural Network (ANN) can be used to build an identification model that would be able to identify tea-grade level. The results showed high precision and considerable accuracy, in addition to reliability, but, unfortunately, they consume a lot of time, making them destructive and unsuitable for online monitoring [258].

#### 5.2.2. Color, Grain Shape, and Size

They can be accessed via organoleptic methods exemplified by human sensory panel, as well as analytical instruments, but these methods consume time, are tedious, have a high cost, and reveal a high variability. Thus, the technology of computer vision was adopted in an effort to combat the previously mentioned drawbacks. This technology aims to duplicate the human vision via electronic perception, which is an automatic process that causes no destruction and is cost-effective. It can be used for the differentiation of tea brands, classes, and colors, as well as in accordance to their grades and discrimination of tea grains under different illumination conditions [261]. Image information of green tea is able to evaluate its sensory quality, which can be predicted by the leaf temperature and moisture content measured during production [262]. 

Back propagation multilayer perceptron (BP-MLP) and radial basis function (RBF) neural networks, along with image information technology, can effectively predict and evaluate the sensory quality of needled green tea, a kind of premium-quality green tea popular in China with an elegant appearance, as changes in leaf temperature and moisture content during green tea processing can affect the content of tea quality ingredients [263] and thus affect the sensory quality of the product [262]. 

### 5.3. Chemical Markers

#### 5.3.1. Catechins

The catechins index (CI) can be used to estimate the quality of green tea owing to the closeness between the quality of green tea and its main catechins contents [10]. The catechin concentration of green tea infusions prepared using purified water is higher than those prepared using mineral water or tap water, as using high-conductivity water reduces the efficiency of catechins extraction, and high pH influences their stability, so reducing the pH of mineral water partially increases the concentration of catechins in the infusions [259]. Recently, a carbon paste working electrode modified by electropolymerizing methylene blue was used for detecting and quantifying catechin concentrations in green tea [264]. In addition, the combined analytical approach using UV-Vis spectrophotometry, matrix-assisted laser desorption/ionization-time-of-flight-mass spectrometry (MALDI-TOF-MS), and inductively coupled plasma-mass spectrometry (ICP-MS) is suitable for the profiling and quality control of green tea via using the polyphenolic fraction (e.g., galloylated flavonoids) as the authentication marker [233].

#### 5.3.2. Caffeine

Additionally, caffeine constitutes a determinant factor in assessing the quality level of green tea. Official green tea was supposed to contain at least 2% of caffeine calculated for the dried substance, as specified by the French Pharmacopoeia [265]. 

#### 5.3.3. Minerals

Great differences in the mineral content (Al, Ca, Mg, and Mn) in green tea from different origins were observed [266]. However, no clear variations were detected between green and black teas regarding the levels of Al, Ba, Ca, Cu, Fe, K, Mg, Mn, Na, Sr, Ti, and Zn [267]. However, a great variation was observed concerning the levels of fluoride and aluminum in various tea varieties, as green tea has lesser Al and F concentrations than black tea [268,269]. 

Additionally, Inductively Coupled Plasma Atomic Emission Spectroscopy (ICP-AES) is used to determine the metal content in different tea samples, and is thus used as a method for its quality control. This was applied in a study done in Saudi Arabia on 17 black tea samples and could effectively differentiate between them. The lowest concentration of Pb was found in Abu Jabal tea; meanwhile, the highest concentration was detected in Manasul tea. The highest Cd level was found in Al-Diafa tea; the Cd level is greatly used to differentiate between black and green samples [270]. 

A physical fractionation of the previously mentioned minerals in green tea infusion was done and used to classify the infusions of different green teas, using chemometric techniques, such as PCA and LDA [271]. However, default transition rates for certain metals, including aluminum, arsenic, cadmium, copper, lead, and mercury, should exist in green tea samples (*C. sinensis*) from different origins, leaf grades, and manufacturing techniques in the dry product and in the infusion, to avoid overestimation of exposure levels upon consumption [272].

#### 5.3.4. Amino Acids

High-grade teas are found to be richer in amino acids in comparison to low-grade ones [273]. Hence, the amount of amino acids in the green tea infusions, especially theanine, which is highly related to the quality evaluation of green tea, should be carefully monitored. Theanine can be measured by a simple, fast, and very convenient method like an enzyme sensor, which consists of l-amino acid oxidase and an oxygen electrode [241]. Meanwhile, a turn-on fluorescent sensor, based on Hg^2+^ coordination conjugated polymer, is able to recover cysteine in several green tea drinks [274]. 

#### 5.3.5. Carotene

It was also found that all quality parameters of tea are enhanced by the increase in endogenous carotene content, which is highly stable in tea due to the existence of antioxidants such as polyphenols and catechins [244]. 

### 5.4. Antioxidant Capacity and Determination of Antioxidant Constituents in Green Tea

The total antioxidant capacity of a green tea extract is an important quality criterion. It can be evaluated via a decolonization assay like the Trolox equivalent antioxidant capacity (TEAC) method, in which the antioxidant capacity is expressed as the concentration of a Trolox solution with equal antioxidant capacity [275]. The antioxidant capacity of green tea infusions prepared using purified water is higher than those prepared using mineral water or tap water [259]. The quantification of the relative antioxidant activities of tea leaves can be made through a direct method like Electron Paramagnetic Resonance (EPR) spectroscopy [276], while Electron Spin Resonance Spectroscopy (ESR) was used directly to measure the level of free radicals, as a marker of early events in oxidation of low-fat dried products such as potato flakes, which can be protected by natural antioxidant green tea extracts [277]. 

ESR and peroxalate chemiluminescence detection system were also used to determine the total catechin levels in green tea through the identification of H_2_O_2_ generated by catechin group, (+)-catechin (CC), EGCG, ECG, and gallic acid, in basic solution [278]. Recently, a zinc oxide fetched lossy mode resonance-based fiber optic biosensor can be used for the determination of H_2_O_2_ production by green tea that is rich in polyphenol [279], and a highly sensitive voltammetric sensor based on a carbon paste electrode with a CuFe_2_O_4_ nanoparticle can be used for the assay of H_2_O_2_ in green tea [280].

Furthermore, the identification and quantification of catechins, gallic acid, and caffeine in green tea extracts was achieved using a rapid reversed-phase HPLC-MS method adopting a gradient elution system with EGCG and EGC as indices of the antioxidant quality of tea extracts [281]. Besides that, five major catechins (EGCG, EGC, ECG, EC, and C) and caffeine in green tea leaves were assessed simultaneously by HPLC, which was effectively used to determine the quality level of green tea concomitantly, with a new chemical pattern recognition tool, Support Vector Classification (SVC), to develop an identification model [10]. Moreover, ultrahigh performance liquid chromatography (UPLC) coupled with UV was adopted for the rapid quantification of green tea catechins, as well as caffeine [282]. UPLC coupled with triple-quadrupole tandem mass spectrometry (HPLC–MS–MS) in multiple-reaction monitoring (MRM) mode was also used to analyze catechins present in green tea simultaneously [283]. 

Tea quality assessment using an electro-analytical technique based on a flow injection analysis coupled with dual electrochemical detector system was used for the highly selective simultaneous detection of polyphenolic functional groups in catechins, especially in EGCG [284]. A modified electrode with high selectivity to catechol can be used to determine polyphenol [285], while gold nanoparticles decorated on porous aromatic framework can be used to directly determine quercetin in green tea samples [286]. 

NIR spectroscopy is a spectroscopic method that uses the near-infrared region of the electromagnetic spectrum, ranging from 780 to 2500 nm. It was recently adopted as an advanced method for assessing the quality of green tea [261]. It is worthy to highlight that NIR spectroscopy needs reference measurements to construct the calibration model by chemometric tools, so analyses of large amounts of similar samples can be made in a few minutes, and even in a few seconds, if the most recent instruments are used. NIR spectroscopy, combined with an appropriate preprocessing method like Standard Normal Variate (SNV), followed by derivation of the spectra in addition to Partial Least Square (PLS) regression, was proved to be an effective method for the prediction of certain quality parameters of green tea, including caffeine content and total antioxidant capacity from the NIR spectra of the entire tea leaves, the total antioxidant capacity from the ground tea, and the EGCG content, but not the EC content, which may be due to its low concentration in the tea [265]. NIR can be used together with PLS algorithm for simultaneous measurement of the main four catechins (EGCG, EGC, ECG, and EC) contents in green tea [287]. 

However, because of the highly complex composition of green tea, the approach of measuring certain parameters is neither appropriate nor sufficient and could be replaced by the information originated from the entire characteristic profile, which is called a “fingerprint” [288]. 

A fingerprint can be obtained by spectroscopic and/or chromatographic techniques [288], such as HPLC, GC, and CE. Chromatograms can give qualitative and quantitative information of the existing compounds that can be quickly retrieved, if required [289]. Fingerprint chromatograms are developed and compared to that of a standardized extract to achieve authentication, identification, and quality control of the green tea, and, in combination with TEAC assay, it permits a good evaluation of the quality of a tea extract [275]. 

Nowadays, the multi-sensors technique represents a new analytical tool that was recently adopted and showed high effectiveness in discrimination, classification, and quality control, along with many advantages, such as being a rapid, low-cost analytical tool relative to HPLC and GC [290]. 

A visual evaluation cannot discriminate between lots of profiles; therefore, mathematical data-handling techniques are recommended [288]. The influence of the experimental variability, including instrumental repeatability, precision, and the variation in sample preparation, together with data preprocessing methods on the ability of the multivariate data analysis methods to discriminate between samples (as well as a new algorithm based on linear regression that was used in the assessment of the quality of green tea extracts) all need to be taken into account [291]. Multivariate analysis was done using by UPLC–quadrupole time-of-flight mass spectrometry (UPLC–Q-TOF/MS) for metabolomics profiling and thus acts as a model for its quality control [292]. 

### 5.5. Determination of Green Tea Contaminants

Nowadays, there is a great interest in tea safety, because contaminants negatively deteriorate human and animal health, as well as cause lots of damage to the environment, resulting in great economic losses. ELISA and immunochemical methods are particularly suitable for ensuring tea safety, owing to their rapidity, simplicity, sensitivity, and low price. The directly suspended droplet micro-extraction method has a high enrichment factor, excellent selectivity, good linearity, and reasonable recovery. Recently, it was found that a hybrid ZnO/g-C_3_N_4_ fiber can be used for simultaneous determination of nine pesticide residues in green tea [293]. 

In addition, an automated multi-plug filtration cleanup medial prefrontal cortex method on modified quick, easy, cheap, effective, rugged, and safe extracts is used to determine pesticide residues in green tea [294]. These include hymexazol and fungicide residue in foods like green tea that could be coupled with liquid chromatography tandem–mass spectrometry [295]. Meanwhile, GC–MS can be used to quantify polycyclic aromatic hydrocarbons as a toxic contaminant in green tea [296]. Using the attenuated total reflection-FTIR (ATR-FTIR) spectroscopic technique in combination with chemometrics techniques, such as HCA and PCA techniques, for the detection of sibutramine in green tea can be achieved [297]. 

However, the combined analytical approach using UV-Vis spectrophotometry, matrix-assisted laser desorption/ionization-time-of-flight-mass spectrometry (MALDI-TOF-MS), and inductively coupled plasma-mass spectrometry (ICP-MS) are suitable for measuring the content of toxic elements, such as Cu, Sr, As, Co, Cr, Fe, Zn, Li, Ba, and Pb, relative to their permitted and recommended limits [233]. Moreover, an automated sequential injection (SI) with second order light scattering (SOS) detection can be used to determine GABA quantity in green tea samples [298]. So many kinds of famous green teas can be identified effectively using the “turn-off” model of quantum dots combined with chemometrics [299].

## 6. Current Trends in Green Tea in Researches and Future Recommendations

Green tea represents about 20% of the dried tea which is manufactured annually and is mainly consumed in Asian countries, such as Japan, owing to its relative safety and cheaper price in comparison to other beverages. Aside from that, it is well-known for its health benefits comprising its anticancer, anti-oxidant, and antimicrobial activities, effectiveness in reducing body weight, and possession of different classes of active constituents, represented by polyphenols, xanthine bases/purine alkaloids, triterpenoid saponins, amino acids, minerals, and trace elements. The beneficial health effects of green tea ultimately lead to its massive consumption and increase its liability to be adulterated by either low-quality or non-green-tea products with a concomitant decrease in activity. 

Additionally, a lot of varieties of tea exist depending upon the different processing techniques represented by green tea, yellow tea, black tea, and white tea, as well as oolong tea. Consequently, the metabolic profile of tea, as well as its biological activity, would drastically change according to the multiple variations between different varieties, as well as due to environmental effects, processing procedure, and the mode of preparation, which ultimately reflect on its quality. Hence, more research aiming to develop advanced, high-quality products to satisfy the diverse demand is needed. This could be achieved via enhancing the efficient use of tea resources, improving both the research and the development level of the different varieties of tea.

New derivatives of teas with a higher quality and more secondary metabolites should be targeted that can be utilized in the form of tea food, tea drinks, tea crafts, tea health products, and tea cosmetics. Undoubtedly, this will require a special focus on improving the quality of these products through the use of simple and available technical methods. Concomitantly, promotion of tea culture should be promoted using various events, like Global Tea Day, that ultimately improve consumers’ recognition of high-quality tea all over the globe. Besides, this will ensure the consumer knowledge about the origins, flavors, and history of the tea they are drinking so that the consumer can act as a driving force for the factories to improve their quality via exploring more advanced but simple techniques in monitoring tea quality and preventing its adulteration worldwide. Moreover, more attention should be given to young consumers and researchers, who are becoming more aware of the beneficial effects of drinking high-quality tea in contrast to drinking adulterated low-quality types. Thus, modern investigations, as well as research, should be conducted to meet their demand with more efforts to be given to find out other active constituents and simple and cheap techniques for its quality assurance that ascertain the prevention of its adulteration and the ease of its incorporation in pharmaceutical industries. Many tea products should be carefully considered; for instance, kombucha tea, which is a type of sugared tea obtained by fermentation by using two types of bacteria, namely acetic acid bacteria and yeast (tea fungus), is known worldwide owing to its massive beneficial and refreshing potentials. It was found to be effective in the prohibition of many types of cancer, in addition to the alleviation of cardiovascular disorders, enhancement of liver functions, and probing of the immune system; thus, special consideration should be given to kombucha tea [300]. 

## 7. Conclusions

Green tea represents about 20% of the dried tea which is manufactured annually and is mainly consumed in Asian countries, such as Japan, owing to its relative safety and cheaper price comparable to other beverages. Besides, it is greatly familiar by its marked health benefits comprising its anticancer, anti-oxidant, antimicrobial activities, effectiveness in reducing body weight, and possession of different classes of active constituents, represented by polyphenols, xanthine bases/purine alkaloids, triterpenoid saponins, amino acids, minerals, and trace elements. The beneficial health effects of green tea ultimately lead to its massive consumption and increase its liability to be adulterated by either low-quality or non-green tea products with concomitant decrease in activity. More studies need to be conducted to find out other biological activates, active constituents, and other simple and cheap techniques for its quality assurance that ascertain the prevention of its adulteration and the ease of its incorporation in the products of pharmaceutical industries.

## Supplementary Materials

The following are available online at https://www.mdpi.com/2076-3921/8/10/455/s1. Table S1: Summary of the major biological activities and mechanism of action of different parts, extracts as well as compounds obtained from green tea (*Camellia sinensis*).
