# Supplementary material for: A Comprehensive Insight on the Health Benefits and Phytoconstituents of Camellia sinensis and Recent Approaches for Its Quality Control
Source: Antioxidants (Basel). 2019 Oct 6;8(10):455. doi: 10.3390/antiox8100455 (PMC6826564; doi:10.3390/antiox8100455)
Supplement: Supplementary file 1 [file antioxidants-08-00455-s001.pdf]

Supplementary Data
Table S1
Summary of the major biological activities and mechanism of action of different parts, extracts as well as compounds obtained from green tea (*Camellia sinensis)*

Activity	Effect	Part Used/Compound/Formulation/Method of consumption or usage	References
**Antioxidant & Hepatoprotective**	↑ Free radical scavenging<br>↓ Lowered GSH<br>↑ Carcinogens excretion	Green tea polyphenols	[1], [2], [3], [4], [5], [6]
	↑ Phase II enzymes	Aqueous extract	[7]
	↓ Oil oxidation	Dechlorophyllized extract	[8]
	↓ Oxidative stress<br>↑ Physical performance	Green tea extract ice cream (Polypenol)	[9]
	↑ Salivary antioxidants	Consumption (Smokers)	[10]
	↓ Cell death	EGCG & (-)-Epigallocatechin-3-(3”-Omethyl) gallate	[11]
	↓ Free radicals produced during metabolism of xenobiotics in the liver	Consumption	[12]
	↓ Fat storage in the liver	Short-time decoction (Chronic usage)	[13]
	↓ Elevated serum ALT, AST, hepatic protein carbonyls & ROS	Leaves	[14]
	↓ Inflammation & hepatic mitochondrial DNA damage	Extract	[15], [16]
	↓ Cisplatin-induced intestinal and hepatic toxicity	Consumption	[17]

**Anti-Cancer & Anti-Mutagenic**	↑ NK cell activity	Flavonoids	[18]
	↑ Apoptosis & tumor suppression<br>↓ Sister-chromatid exchange	Polyphenols	[19], [20], [21]
	↓ Angiogenesis<br>↓ MMPs	Catechins	[22], [23], [24]<br>[25], [26]
	↑ Gap junctional intercellular communication<br>↓ FAS activity<br>↓VEGF Family	EGCG & its auto-oxidation products	
	↑ cAMP	Methyl xanthines	[27]
	↑ Macrophages and NK cells activity	Polysaccharides	[28]
	↑ Metabolism & Excretion of carcinogens	Aqueous extract	[29], [30], [31], [32], [33], [34], [35], [36].
	↑ Fidelity of DNA replication<br>↓ TNF-α gene expression	EGCG	[37], [38], [39].
	↓ Microsomal aryl hydrocarbon hydroxylase activity<br>Antigenotoxic activity	(+)catechin, EC, EGC, ECG & EGCG	[21].
	Late onset of cancer & ↓ RR of its incidence	Pheophytins a and b	[34].
	Chemopreventive agent	Increased consumption	[40].
	↓ O_2^- & NO production	↑ EGCG:Caffeine	[41].
	↑ UDP-glucuronosyltransferase induction	Catechin	[42].

	↑ Toxin metabolism	Consumption	(43).
	↑ P-glycoprotein-mediated efflux function	Catechin, EC, ECG, gallocatechin, EGC & EGCG<br>EC	(44)<br>(45)
**Skin**	Anti-tumor promotion<br>↓ Tumorigenicity<br>↓ Induced inflammatory Rx<br>↓ Skin lesions & redness<br>↓ Epidermal microsomes lipid peroxidation<br>↓ Photocarcinogenesis<br>↓ Tyrosinase activity	EGCG<br>Tannic acid & GTPs<br>Pheophytin a and b<br>Oral consumption<br>EGCG>ECG>EGC>EC<br>EGCG<br>ECG, GCG & EGCG	(46)<br>(47)<br>(48)<br>(49), (50)<br>(51)<br>(52)<br>(53)
**Forestomach**	Interaction & ↓ metabolism of carcinogen<br>↓ Metastasis	GTPs	(53), (54), (55)
**Duodenum**	↓ Duodenal cancer	EGCG	(56)
**Colon**	↓ Promotion stage<br>↓ Incidence & number<br>↓ Colonic mucosa DNA damage<br>↓ Metachronous adenomas incidence<br>↓ LOX- & COX- dependent arachidonic acid metabolism<br>↓ Colorectal carcinoma cells<br>↓ Topoisomerase I activity	GTPs<br>Extract<br>ECG> EGCG & EGC	(57)<br>(58), (59),(60), (61)<br>(62)<br>(63)

	& ↓ Intermediate & late stages	EGC & EGCG < EC<br><br>EGCG	(64), (65)
**Lung**	↓ Mortality<br><br>↓ Mean number & ↓ multiplicity of adenomas<br>↑ Superoxide radicals scavenging<br>↓Lung cancer stem cells<br>↑ Apoptosis<br><br>↓ Penetration of cancerous cells through the basement membrane<br><br>↓ Cells DNA strand breaks<br><br>↑ Apoptosis<br><br>↓ Invasion of highly metastatic cancerous cells	Consumption<br><br>EGCG<br><br><br><br><br><br>Infusion & Catechins<br><br><br><br>GTPs<br><br>EGCG & EC<br><br>ECG	(66).<br><br>(67), (68), (69), (70).<br><br><br><br><br><br>(71).<br><br><br><br>(72).<br><br>(73).<br><br>(74).
**Leukemia**	↓ Sister-chromatid exchanges<br><br>↓ Growth factors' signaling, ↓ differentiation, ↑ proliferation & ↑ B-cell and T-cell chronic lymphocytic leukemia apoptosis<br><br>↓ Acute Myeloid Leukemia	Crude catechin<br><br>EGCG<br><br><br><br>Consumption	(75).<br><br>(76), (77), (78).<br><br><br><br>(79).
**Breast**	↓ Mortality, ↓ average sizes of tumors & ↓ post-initiation stage<br><br>↓ Early promotion stage<br><br>↓ Stages I and II recurrence rate	Catechins<br><br><br><br>Polyphenon E (58.4% EGCG)	(80).<br><br><br><br>(81),(82).

	↓ Early stage recurrence	High daily consumption	[73].
	↓ PSA production	Regular consumption	[83].
	↓ Ornithine decarboxylase	Flavonoids	[84].
	↓ Cell growth & ↑ cell death	GTPs	[84].
	↓ Cell cycle progression<br>↓ Size & number of tumors	Extract	[85].
	↓ Human breast cancer	EGCG	[86], [87].
		Selenium-enriched GTP	[88].
**Large intestine**	↓ Number & size of cancers	GTPs	[3].
	↓ Iκ-B kinase complex activity	EGCG	[89].
**Liver**	↓ Oxygen free radical-generated cell death<br>↓ inhibition of gap junctional intercellular communication	Catechins	[90].
	↓ DNA damage & ↓ cell proliferation<br>↓ Hepatotoxicity	Crude catechin	[91].
	↓ Tumor number	Decaffeinated intake	[67].
	↓ NOS activity	Polyphenols	[92].
	↓ Hepatocellular carcinoma cells cancer growth	EGC & EGCG >EC	[63].
**Ascites**	↓ Doxorubicin efflux	Theanine	[93].

	↓ Tumor cells viability	EGC & Extract	[94], [95].
**Prostate**	↓ Cancer cell growth & ↑ Apoptosis<br><br>↑ Cancer-free cases<br><br>↓ PSA production<br><br>↓ Cell growth & ↑ Apoptosis<br><br>↓ Advanced prostate cancers risk	EGCG<br><br>GTP oral infusion (6 cups/day)<br><br>Flavonoids<br><br>Extract & ECG > EGCG > EGC<br><br>Intake	[96], [97], [98], [99].<br><br>[100].<br><br>[84].<br><br>[101].<br><br>[102].
**Ovary**	↓ Adriamycin efflux<br><br>↓ Risk	Theanine<br><br>Consumption	[103].<br><br>[104].
**Bladder**	↓ Cell proliferation & viability, ↑ apoptosis & ↓ cancer cells migration & tumor growth	EGCG	[105], [106].
**Brain**	↓ Glioblastoma cells<br><br>↓ inflammatory processes	Catechins gallate & EGCG<br><br>EGCG	[107].<br><br>[108], [109].
**Kidney**	↓ Growth	Intake, Extract & EGCG	[110], [111].
**Cervix**	↑ Apoptosis	EGCG > Polyphenol E	[112].
**Head & Neck**	↓ Progression	EGCG	[113].
**Papillomavirus-induced neoplasia**	↓Neoplasia	EGCG	[114].
**Antimicrobial**	↑ Bacterial membranes damage & ↑ morphological distortions of *Candida*<br><br>↓ Extracellular vero toxins	Catechins	[115], [116].

	production and release	EGCG & GCG	[117].
	↓ *Salmonella typhimurium* type III activity & invasion, ↓ ricin toxin, ↓ Brazilian strain of Zika virus entry & ↓ efflux pumps of carbapenem	EGCG	[118], [119], [120], [121].
	Antiseptic, ↓ several pathogenic bacteria activity, ↓ bacterial spores development & growth, ↓ bacteriophages	Polyphenols	[1], [122], [123].
	Bactericidal against G+ve, Bacteriostatic against G-ve, less anti-fungal, ↓ Dental caries bacteria, ↓ facultative anaerobes, ↓ obligative anaerobes	Extract	[124], [125], [126].
	↓ 1-Deoxy-D-xylulose 5-phosphate reductoisomerase activity<br>↑ Apoptotic markers<br>↓ Multidrug resistant bacterial strain activity	Raw extract (GCG)	[127].
	↓ HIV sexual transmission	Chlorogenic acid	[128].
	↓ HIV-1 Infectivity	GA & Polyphenols	[129].
		EGCG (Topical)	[130].
		Polyphenon E (EGCG drug formulation)	[131].
**Anti-Schistosomiasis & Anti-Parasitic**	↑ Hepatocytes improvement, ↓ cellular necrosis & ↑ total protein & glycogen levels<br>Anti-parasitic activity	Aqueous extract	[132], [133].

**Cardioprotective**	↓ Risk factors for heart disease	Consumption	[134].
	↓ Coronary heart disease development	Non-fermented Beverage & Catechin-rich fractions	[135].
	↑ Vascular function	High pressure process prepared	[136].
	↓ CV risk factor	*O*-methylated catechin	[137].
	↓ Myocardial fibrotic degeneration	Catechins	[138].
	↓ CV diseases risk & ↓ Coronary atherosclerosis	Consumption (7-8 cups /day)	[139], [140], [141].
	↓ MI		
	↓ CAD risk	Consumption (>1 cup/ day)	[142].
	↓ Cardiac injury following ischemia ↑ Coronary flow velocity reserve	Highest consumption	[143], [144].
	Adjunct therapy of CV disease	High dietary intake	[145].
	↓ Doxorubicin-induced modifications in cardiomyocyte fatty acid composition	Flavonoids	[146].
	↑ Excretion & ↓ Absorption of cholesterol	Polyphenols	[147].
	↓ Superoxide anion generation & ↓ High BP	Extract	[148], [149], [150].
	↓ Doxurubicin-induced cardiotoxicity, ↓ arsenic-induced cardiotoxicity, ↓ inflammation & mortality rate		
	↓ Cardiac arrhythmia, ↑ fasting plasma insulin, ↑ insulin resistance		[151], [152], [153].

		EGCG	
	↓ LDL oxidation		
	↓ Biosynthesis		
			(154),(155).
	↓ LDL lipid peroxidation	EC	
	↓ Biogenesis		
			(156).
	↓ Free radicals-induced LDL peroxidation	Consumption	
			(157).
		Processed & Unprocessed extract	
			(158).
	↑ α-tocopherol regeneration		
		Polyphenols > Caffeine	
			(159).
	↓ Endothelial cell-induced LDL oxidation	EGCG, GCG & ECG	
			(160).
	↑ LDL resistance to *in vivo* oxidation	EC>EGCG>ECG>EGC> GA	
			(160).
	↓ LDL		
		ECG, EC, EGCG, EGC & GA	
			(135).
	↑ LDL oxidation lag time		
		Catechin-rich fractions	
	↓ Serum TGs		
			(139).
	↓ Fat storage in the liver & ↓ blood lipids and ↑ TGs fecal excretion	Daily consumption of 7-8 cups	
			(161).
	↓ Dyslipidemia	Daily consumption of ≥ 10 cups	
			(162).
	↓ High BP	Flavonoids	
			(163).
		Floratheasaponins A-C (Flowers>Seeds)	
			(13).
	↓ Dopa decarboxylase enzyme		
		Short-time decoction (Chronic usage)	
	↓ ROS		
	↓ ROS formation	*O*-methylated catechin	(137).
	↓ Thromboxane-B2	Fresh leaves fermented under N2 gas & Theanine	(164)

	↓ Thrombosis	EGCG & EGC	(165). (166).
	↓ Sickle cells dehydration & ↑ Dense cells formation	Chronic infusion of EGCG in PVN	(167).
		Decaffeinated extract	(168).
		Unprocessed extracts	(157).
		Catechins & EGCG	(169).
		Extracts (↑ OHs group)	(170).
**Anti-Diabetic & Anti-Obesity**	↑ Glucose tolerance, ↓ elevated serum glucose level, ↓ elevtated hepatic and renal enzymes, ↓ elevated serum lipid peroxidation levels, ↑ decreased liver glycogen, ↑ GSH levels & ↓ endothelial autophage inhibition "Exercise mimetic" prosperities ↓ Body weight	GTPs	(171), (172), (173), (57).
	↓ Endothelial cell growth, ↓ diabetes complications & ↑ insulin-resistant muscle resensitization ↑ Lipolysis, ↓ FAS activity, ↑ energy expenditure & ↑ metabolic rate↓ Serum glucose elevation ↓ α-amylase activity ↓ pancreatic α-amylase activity	EGCG	(174), (175), (176), (177), (178), (26), (179).
	↑ Glucose and lipids metabolism & ↓ insulin consumption	Floratheasaponins A, B, and C	(180).
	↓ Fasting glucose in elderly	H2O-soluble polysaccharide fraction	(181).

	↓ Waist circumferences<br><br>↓ Hyperglycemia<br>↓ Fluid consumption, ↓ body weight & ↓ waist circumference<br><br>↓ Fat storage in the liver & ↓ glucose<br><br>↑ Fasting plasma insulin & ↓ insulin resistance<br><br>↓ Fat digestion<br><br>↑ Thermogenesis, ↑ fat oxidation & ↓ Adipogenesis<br><br>↓ Obesity<br><br>↓ Weight gain<br><br>↓ Average body weight<br><br>↑ Energy expenditure, & ↑ fat oxidation<br><br>↓ Weight gain<br><br>↓ Weight & body mass index	Catechin (Galloyl moiety)<br><br>Consumption<br><br>Green tea-Vit. E (Daily)<br><br>Extract<br><br>Short-time decoction (Chronic usage)<br><br>EC<br><br>Saponins<br><br>EGCG & Caffeine<br><br>High pressure process prepared<br><br>Caffeine<br><br>Catechol & TA<br><br>Catechins & Caffeine<br><br>Short-time decoction (Chronic usage) & EGCG<br><br>Consumption (with training)	[182].<br><br>[183].<br><br>[184].<br><br>[185], [186], [187].<br><br>[13].<br><br>[155].<br><br>[188].<br><br>[189], [173].<br><br>[136].<br><br>[190].<br><br>[80].<br><br>[191].<br><br>[13].<br><br>[192].

**Gastrointestinal Tract Problems Relieving**	↑ Mesenteric arteries relaxation<br><br>↑ Elastin content regeneration, ↓ inflammation & ↓ abdominal aortic aneurysm<br><br>↓ Precancerous chronic atrophic gastritis<br><br>↓ Gastric mucosal lesions<br><br>↓ *Clostridium difficile*<br>↓ *Clostridium perfringens*<br><br>Faecal microbiome & endogenous metabolites modulation	EC<br><br>EGCG<br><br>High consumption<br><br>Floratheasaponins A, B, and C<br><br>Vitamin C<br>Polyphenols<br><br>Consumption	(193).<br><br>(194).<br><br>(195).<br><br>(180).<br><br>(196).<br><br>(197).
**Neuroproetctive**	↓ Lipid peroxidation damage<br><br>↓ Stress-induced neural injuries<br>↓ Ischemic neuronal damage & ↓ Increase of nitric oxide radical concentrations in the intact hippocampus<br>↑ Angiogenesis<br>↓ TH-positive cells loss, ↓ nNOS expression, ↓ neuronal apoptosis & ↑ growth and survival of astrocytes regulation<br>↑ Spatial memory function in mice induced-dementia<br><br>↓ Ischemia/reperfusion-induced brain injury<br>↓ Age-related deficits, ↓ cognitive decline, ↓ stroke-induced short & long term declarative memory	EGCG, ECG, EGC & EC<br><br>EGCG<br><br>Extract	(198).<br><br>(199), (200), (201), (202), (203), (204), (205),(206), (207).<br><br>(208), (209), (210), (211), (212).

	Stroke onset prevention	Consumption (3 cups/ day)	[213].
	↓ Stroke risk	Consumption (>3 cups/ day)	[140],[161].
	↓ Neurotoxicity<br>↑ Memory & ↑ learning ability<br>Affecting Emotions<br>↑ Relaxation	L-theanine	[214], [165].
	Neuroprotective	Polyphenols	[215], [216].
	↓ Morphologic & functional changes in accelerated senile brains & ↓ β-secretase activity	Catechin	[217], [218].
	↓ Dopa decarboxylase activity	EGCG & EGC	[166].
	↓ Amyloid-β aggregates	EC	[219].
	↑ Learning ability	EGC & GA ingestion > EGCG	[220].
	Neuronal activity modulator	Polyphenols (EGCG)	[221].
	Synaptic transmission modulator	Consumption	[222].
	↓ Adrenal hypertrophy (Caffeine & EGCG)	Theanine (low caffeine), EGC & arginine	[223].
**Anti-Inflammatory, Analgesic, Anti-Pyretic & Anti-Allergic**	↓ Inflammatory diseases	Consumption	[224], [225].
	↓ NO production<br>& ↓ Angiogenesis	EGCG	[226], [174].
	↓ PMNs activation	Pheophytin a and b	[48].
	Anti-pyretic & Diuretic	Consumption	[227].
	↓ Pain	Extract & Tablets	[228], [229], [230].

	↓ Histamine release	Polyphenols	[231].
	↓ Allergy	Leaf Saponins & Floratheasaponins	[232], [233].
	↓ Histamine & IgE production	Methylated EGCG	
	↑ Heat-generating mechanisms	Consumption	
**Skeletomuscular System Relieving**	↑ Skeletal muscles vasodilatation, ↑ muscle microvascular blood flow, ↑ myogenic differentiation & ↑ muscles mass & function recovery<br>↑ Osteoclasts cell death, ↓ Inflammation, ↑ Bone regeneration, ↑ osteoprotegerin synthesis & ↓ osteoclastic bone resorption<br>↓ NO production & lactate dehydrogenase release<br><br>↓ Bone and hyaline cartilage alteration	EGCG	[234], [235], [236], [237], [238], [239], [240].
	↓ inflammation & joint destruction	Consumption	[39]
	↓ Pain & ↑ knee joint physical function	Consumption (EGCG)	[39].
		Extract & Tablets	[229], [230].

References

1. Kerr, C.,Venter, A. In brief. Trends in Microbiology. **2002**, *10* p. 312-313.

2. Barroso, M.F.;Ramalhosa, M.J.;Alves, R.C.;Dias, A.;Soares, C.M.D.;Oliva-Teles, M.T., Delerue-Matos, C. Total antioxidant capacity of plant infusions: Assessment using electrochemical DNA-based biosensor and spectrophotometric methods. Food Control. **2016**, *68* p. 153-161.
3. Pingzhang, Y.;Jinying, Z.;Shujun, C.;Hara, Y.;Qingfan, Z., Zhengguo, L. Experimental studies of the inhibitory effects of green tea catechin on mice large intestinal cancers induced by 1, 2-dimethylhydrazine. Cancer Letters. **1994**, *79* p. 33-38.
4. Zhao, B.;Guo, Q., Xin, W. Free radical scavenging by green tea polyphenols. Methods in Enzymology. **2001**, *335* p. 217.
5. Lee, S.F.;Liang, Y.C., Lin, J.K. Inhibition of 1, 2, 4-benzenetriol-generated active oxygen species and induction of phase II enzymes by green tea polyphenols. Chemico-Biological Interactions. **1995**, *98* p. 283-301.
6. Vermeer, I.T.;Moonen, E.J.;Dallinga, J.W.;Kleinjans, J.C., van Maanen, J.M. Effect of ascorbic acid and green tea on endogenous formation of N-nitrosodimethylamine and N-nitrosopiperidine in humans. Mutation Research/Fundamental and Molecular Mechanisms of Mutagenesis. **1999**, *428* p. 353-361.
7. Bu-Abbas, A.;Clifford, M.;Walker, R., Ioannides, C. Contribution of caffeine and flavanols in the induction of hepatic phase II activities by green tea. Food and Chemical Toxicology. **1998**, *36* p. 617-621.
8. Wanasundara, U.N.,Shahidi, F. Antioxidant and pro-oxidant activity of green tea extracts in marine oils. Food Chemistry. **1998**, *63* p. 335-342.
9. Sanguigni, V.;Manco, M.;Sorge, R.;Gnessi, L., Francomano, D. Natural antioxidant ice cream acutely reduces oxidative stress and improves vascular function and physical performance in healthy individuals. Nutrition. **2017**, *33* p. 225-233.
10. Azimi, S.;Mansouri, Z.;Bakhtiari, S.;Tennant, M.;Kruger, E.;Rajabibazl, M., Daraei, A. Does green tea consumption improve the salivary antioxidant status of smokers? Archives of Oral Biology. **2017**, *78* p. 1-5.
11. Kagaya, N.;Tagawa, Y.-i.;Nagashima, H.;Saijo, R.;Kawase, M., Yagi, K. Suppression of cytotoxin-induced cell death in isolated hepatocytes by tea catechins. European Journal of Pharmacology. **2002**, *450* p. 231-236.
12. Casas-Grajales, S.,Muriel, P., *Chapter 43 - The Liver, Oxidative Stress, and Antioxidants*, in *Liver Pathophysiology*. 2017, Academic Press: Boston. p. 583-604.
13. Hamdaoui, M.H.;Snoussi, C.;Dhaouadi, K.;Fattouch, S.;Ducroc, R.;Le Gall, M., Bado, A. Tea decoctions prevent body weight gain in rats fed high-fat diet; black tea being more efficient than green tea. Journal of Nutrition & Intermediary Metabolism. **2016**, *6* p. 33-40.
14. Jayashree, G.V.;Krupashree, K.;Rachitha, P., Khanum, F. Patulin Induced Oxidative Stress Mediated Apoptotic Damage in Mice, and its Modulation by Green Tea Leaves (GTL). Journal of Clinical and Experimental Hepatology. **2017**,

15. Li, J.;Sapper, T.N.;Mah, E.;Moller, M.V.;Kim, J.B.;Chitchumroonchokchai, C.;McDonald, J.D., Bruno, R.S. Green tea extract treatment reduces NFκB activation in mice with diet-induced nonalcoholic steatohepatitis by lowering TNFR1 and TLR4 expression and ligand availability. The Journal of Nutritional Biochemistry. **2017**, *41* p. 34-41.
16. Reddyvari, H.;Govatati, S.;Matha, S.K.;Korla, S.V.;Malempati, S.;Pasupuleti, S.R.;Bhanoori, M., Nallanchakravarthula, V. Therapeutic effect of green tea extract on alcohol induced hepatic mitochondrial DNA damage in albino wistar rats. Journal of Advanced Research. **2017**, *8* p. 289-295.
17. AK, S.;Shubha, P.;Sheeba, K.;Wasim, M., Yusufi, A.N. Green Tea Consumption Ameliorates Intestinal and Hepatic-Toxicity induced by Long-Term administration of Cisplatin. International Journal of Drug Metabolism & Toxicology. **2018**,
18. Burkard, M.;Leischner, C.;Lauer, U.M.;Busch, C.;Venturelli, S., Frank, J. Dietary flavonoids and modulation of natural killer cells: implications in malignant and viral diseases. The Journal of Nutritional Biochemistry. **2017**, *46* p. 1-12.
19. Yu, R.;Jiao, J.;Duh, J.;Gudehithlu, K.;Tan, T., Kong, A. Activation of mitogen-activated protein kinases by green tea polyphenols: potential signaling pathways in the regulation of antioxidant-responsive element-mediated phase II enzyme gene expression. Carcinogenesis. **1997**, *18* p. 451-456.
20. Ahmad, N.;Cheng, P., Mukhtar, H. Cell cycle dysregulation by green tea polyphenol epigallocatechin-3-gallate. Biochemical and Biophysical Research Communications. **2000**, *275* p. 328-334.
21. Wang, Z.Y.;Cheng, S.J.;Zhou, Z.C.;Athar, M.;Khan, W.A.;Bickers, D.R., Mukhtar, H. Antimutagenic activity of green tea polyphenols. Mutation Research/Genetic Toxicology. **1989**, *223* p. 273-285.
22. Rashidi, B.;Malekzadeh, M.;Goodarzi, M.;Masoudifar, A., Mirzaei, H. Green tea and its anti-angiogenesis effects. Biomedicine & Pharmacotherapy. **2017**, *89* p. 949-956.
23. Demeule, M.;Brossard, M.;Pagé, M.;Gingras, D., Béliveau, R. Matrix metalloproteinase inhibition by green tea catechins. Biochimica et Biophysica Acta (BBA)-Protein Structure and Molecular Enzymology. **2000**, *1478* p. 51-60.
24. Jha, S.;Kanaujia, S.P., Limaye, A.M. Direct inhibition of matrix metalloproteinase-2 (MMP-2) by (−)-epigallocatechin-3-gallate: A possible role for the fibronectin type II repeats. Gene. **2016**, *593* p. 126-130.
25. Sigler, K.,Ruch, R.J. Enhancement of gap junctional intercellular communication in tumor promoter-treated cells by components of green tea. Cancer Letters. **1993**, *69* p. 15-19.
26. Wang, X.,Tian, W. Green tea epigallocatechin gallate: a natural inhibitor of fatty-acid synthase. Biochemical and Biophysical Research Communications. **2001**, *288* p. 1200-1206.
27. Rall, T., *The methylxanthines. The pharmacological basis of therapeutics*, 1985, New York: Macmillan Publishing Co.

28. Park, H.-R.;Hwang, D.;Suh, H.-J.;Yu, K.-W.;Kim, T.Y., Shin, K.-S. Antitumor and antimetastatic activities of rhamnogalacturonan-II-type polysaccharide isolated from mature leaves of green tea via activation of macrophages and natural killer cells. International Journal of Biological Macromolecules. **2017**, *99* p. 179-186.
29. Bu-Abbas, A.;Clifford, M.;Ioannides, C., Walker, R. Stimulation of rat hepatic UDP-glucuronosyl transferase activity following treatment with green tea. Food and Chemical Toxicology. **1995**, *33* p. 27-30.
30. Rashidinejad, A.;Birch, E.J.;Hindmarsh, J., Everett, D.W. Molecular interactions between green tea catechins and cheese fat studied by solid-state nuclear magnetic resonance spectroscopy. Food Chemistry. **2017**, *215* p. 228-234.
31. Choi, S.Y.;Chung, M.J., Sung, N. Volatile N-nitrosamine inhibition after intake Korean green tea and Maesil (Prunus mume SIEB. et ZACC.) extracts with an amine-rich diet in subjects ingesting nitrate. Food and Chemical Toxicology. **2002**, *40* p. 949-957.
32. Kato, R.;Kamataki, T., Yamazoe, Y. N-hydroxylation of carcinogenic and mutagenic aromatic amines. Environmental Health Perspectives. **1983**, *49* p. 21.
33. Kuroda, Y. Bio-antimutagenic activity of green tea catechins in cultured Chinese hamster V79 cells. Mutation Research/Environmental Mutagenesis and Related Subjects. **1996**, *361* p. 179-186.
34. Okai, Y.,Higashi-Okai, K. Potent suppressing activity of the non-polyphenolic fraction of green tea (Camellia sinensis) against genotoxin-induced umu C gene expression in Salmonella typhimurium (TA 1535/pSK 1002)–association with pheophytins a and b. Cancer Letters. **1997**, *120* p. 117-123.
35. Jiang, T.;Glickman, B.W., de Boer, J.G. Protective effect of green tea against benzo [a] pyrene-induced mutations in the liver of Big Blue® transgenic mice. Mutation Research/Fundamental and Molecular Mechanisms of Mutagenesis. **2001**, *480* p. 147-151.
36. Edenharder, R.;Sager, J.W.;Glatt, H.;Muckel, E., Platt, K.L. Protection by beverages, fruits, vegetables, herbs, and flavonoids against genotoxicity of 2-acetylaminofluorene and 2-amino-1-methyl-6-phenylimidazo[4,5-b]pyridine (PhIP) in metabolically competent V79 cells. Mutation Research/Genetic Toxicology and Environmental Mutagenesis. **2002**, *521* p. 57-72.
37. Kada, T.;Kaneko, K.;Matsuzaki, S.;Matsuzaki, T., Hora, Y. Detection and chemical identification of natural bio-antimutagens: a case of the green tea factor. Mutation Research/Fundamental and Molecular Mechanisms of Mutagenesis. **1985**, *150* p. 127-132.
38. Fujiki, H.;Suganuma, M.;Okabe, S.;Kurusu, M.;Imai, K., Nakachi, K. Involvement of TNF-α changes in human cancer development, prevention and palliative care. Mechanisms of Ageing and Development. **2002**, *123* p. 1655-1663.

39. Singh, R.;Akhtar, N., Haqqi, T.M. Green tea polyphenol epigallocatechi3-gallate: Inflammation and arthritis. Life Sciences. **2010**, *86* p. 907-918.
40. Imai, K.;Suga, K., Nakachi, K. Cancer-Preventive Effects of Drinking Green Tea among a Japanese Population. Preventive Medicine. **1997**, *26* p. 769-775.
41. Baptista, J.A.;da P Tavares, J.F., Carvalho, R.C. Comparative study and partial characterization of Azorean green tea polyphenols. Journal of Food Composition and Analysis. **1999**, *12* p. 273-287.
42. Sarkar, A.,Bhaduri, A. Black tea is a powerful chemopreventor of reactive oxygen and nitrogen species: comparison with its individual catechin constituents and green tea. Biochemical and Biophysical Research Communications. **2001**, *284* p. 173-178.
43. Embola, C.;Sohn, O.;Fiala, E., Weisburger, J. Induction of UDP-glucuronosyltransferase 1 (UDP-GT1) gene complex by green tea in male F344 rats. Food and Chemical Toxicology. **2002**, *40* p. 841-844.
44. Rameshrad, M.;Razavi, B.M., Hosseinzadeh, H. Protective effects of green tea and its main constituents against natural and chemical toxins: A comprehensive review. Food and Chemical Toxicology. **2017**, *100* p. 115-137.
45. Wang, E.-j.;Barecki-Roach, M., Johnson, W.W. Elevation of P-glycoprotein function by a catechin in green tea. Biochemical and Biophysical Research Communications. **2002**, *297* p. 412-418.
46. Yoshizawa, S.;Horiuchi, T.;Fujiki, H.;Yoshida, T.;Okuda, T., Sugimura, T. Antitumor promoting activity of (−)-epigallocatechin gallate, the main constituent of “Tannin” in green tea. Phytotherapy Research. **1987**, *1* p. 44-47.
47. Khan, W.;Wang, Z.;Athar, M.;Bickers, D., Mukhtar, H. Inhibition of the skin tumorigenicity of (k)-7P, 8wdihydroxy-90, IOa-epoxy-7, 8, 9, 10-tetrahydrobenzo [a] pyrene by tannic acid, green tea polyphenols and quercetin in Sencar mice. Cancer Letters. **1988**, *42* p. 7-12.
48. Higashi-Okai, K.;Otani, S., Okai, Y. Potent suppressive activity of pheophytin a and b from the non-polyphenolic fraction of green tea (Camellia sinensis) against tumor promotion in mouse skin. Cancer letters. **1998**, *129* p. 223-228.
49. Conney, A.H.;Wang, Z.-Y.;Huang, M.-T.;Ho, C.-T., Yang, C.S. Inhibitory effect of green tea on tumorigenesis by chemicals and ultraviolet light. Preventive medicine. **1992**, *21* p. 361-369.
50. Record, I.R.,Dreosti, I.E. Protection by black tea and green tea against UVB and UVA+ B induced skin cancer in hairless mice. Mutation Research/Fundamental and Molecular Mechanisms of Mutagenesis. **1998**, *422* p. 191-199.
51. Katiyar, S.K.;Agarwal, R., Mukhtar, H. Inhibition of spontaneous and photo-enhanced lipid peroxidation in mouse epidermal microsomes by epicatechin derivatives from green tea. Cancer Letters. **1994**, *79* p. 61-66.

52. Wei, H.;Zhang, X.;Zhao, J.-F.;Wang, Z.-Y.;Bickers, D., Lebwohl, M. Scavenging of hydrogen peroxide and inhibition of ultraviolet light-induced oxidative DNA damage by aqueous extracts from green and black teas. Free Radical Biology and Medicine. **1999**, *26* p. 1427-1435.
53. Katiyar, S.K.;Agarwal, R., Mukhtar, H. Protective effects of green tea polyphenols administered by oral intubation against chemical carcinogen-induced forestomach and pulmonary neoplasia in A/J mice. Cancer Letters. **1993**, *73* p. 167-172.
54. Wang, Z.Y.;Das, M.;Bickers, D., Mukhtar, H. Interaction of epicatechins derived from green tea with rat hepatic cytochrome P-450. Drug Metabolism and Disposition. **1988**, *16* p. 98-103.
55. Arcone, R.;Palma, M.;Pagliara, V.;Graziani, G.;Masullo, M., Nardone, G. Green tea polyphenols affect invasiveness of human gastric MKN-28 cells by inhibition of LPS or TNF-α induced Matrix Metalloproteinase-9/2. Biochimie Open. **2016**, *3* p. 56-63.
56. Fujita, Y.;Yamane, T.;Tanaka, M.;Kuwata, K.;Okuzumi, J.;Takahashi, T.;Fujiki, H., Okuda, T. Inhibitory Effect of (—)-Epigallocatechin Gallate on Carcinogenesis with N-Ethyl-N′-nitro-N-nitrosoguanidine in Mouse Duodenum. Cancer Science. **1989**, *80* p. 503-505.
57. Yamane, T.;Hagiwara, N.;Tateishi, M.;Akachi, S.;Kim, M.;Okuzumi, J.;Kitao, Y.;Inagake, M.;Kuwata, K., Takahashi, T. Inhibition of Azoxymethane-induced Colon Carcinogenesis in Rat by Green Tea Polyphenol Fraction. Cancer Science. **1991**, *82* p. 1336-1339.
58. Narisawa, T.,Fukaura, Y. A very low dose of green tea polyphenols in drinking water prevents N-methyl-N-nitrosourea-induced colon carcinogenesis in F344 rats. Japanese Journal of Cancer Research. **1993**, *84* p. 1007-1009.
59. Inagake, M.;Yamane, T.;Kitao, Y.;Oya, K.;Matsuraoto, H.;Kikuoka, N.;Nakatani, H.;Takahashi, T.;Nishimura, H., Iwashiraa, A. Inhibition of 1, 2-Dimethylhydrazine-induced Oxidative DNA Damage by Green Tea Extract in Rat. Japanese Journal of Cancer Research. **1995**, *86* p. 1106-1111.
60. Matsumoto, H.;Yamane, T.;Inagake, M.;Nakatani, H.;Iwata, Y.;Takahashi, T.;Nishimura, H.;Nishino, H.;Nakagawa, K., Miyazawa, T. Inhibition of mucosal lipid hyperoxidation by green tea extract in 1, 2-dimethylhydrazine-induced rat colonic carcinogenesis. Cancer Letters. **1996**, *104* p. 205-209.
61. Shin, C.M.;Lee, D.H.;Seo, A.Y.;Lee, H.J.;Kim, S.B.;Son, W.-C.;Kim, Y.K.;Lee, S.J.;Park, S.-H.;Kim, N.;Park, Y.S., Yoon, H. Green tea extracts for the prevention of metachronous colorectal polyps among patients who underwent endoscopic removal of colorectal adenomas: A randomized clinical trial. Clinical Nutrition. **2017**,

62. Hong, J.;Smith, T.J.;Ho, C.-T.;August, D.A., Yang, C.S. Effects of purified green and black tea polyphenols on cyclooxygenase-and lipoxygenase-dependent metabolism of arachidonic acid in human colon mucosa and colon tumor tissues. Biochemical Pharmacology. **2001**, *62* p. 1175-1183.
63. Uesato, S.;Kitagawa, Y.;Kamishimoto, M.;Kumagai, A.;Hori, H., Nagasawa, H. Inhibition of green tea catechins against the growth of cancerous human colon and hepatic epithelial cells. Cancer Letters. **2001**, *170* p. 41-44.
64. Berger, S.J.;Gupta, S.;Belfi, C.A.;Gosky, D.M., Mukhtar, H. Green tea constituent (−)-epigallocatechin-3-gallate inhibits topoisomerase I activity in human colon carcinoma cells. Biochemical and Biophysical Research Communications. **2001**, *288* p. 101-105.
65. Orner, G.A.;Dashwood, W.-M.;Blum, C.A.;Díaz, G.D.o.;Li, Q.;Al-Fageeh, M.;Tebbutt, N.;Heath, J.K.;Ernst, M., Dashwood, R.H. Response of Apcmin and A33ΔNβ-cat mutant mice to treatment with tea, sulindac, and 2-amino-1-methyl-6-phenylimidazo[4,5-b]pyridine (PhIP). Mutation Research/Fundamental and Molecular Mechanisms of Mutagenesis. **2002**, *506–507* p. 121-127.
66. Wynder, E.L.;Fujita, Y.;Harris, R.E.;Hirayama, T., Hiyama, T. Comparative epidemiology of cancer between the United States and Japan. A second look. Cancer. **1991**, *67* p. 746-763.
67. Cao, J.;Xu, Y.;Chen, J., Klaunig, J.E. Chemopreventive effects of green and black tea on pulmonary and hepatic carcinogenesis. Toxicological Sciences. **1996**, *29* p. 244-250.
68. Taniguchi, S.i.;Fujiki, H.;Kobayashi, H.;Go, H.;Miyado, K.;Sadano, H., Shimokawa, R. Effect of (−)-epigallocatechin gallate, the main constituent of green tea, on lung metastasis with mouse B16 melanoma cell lines. Cancer Letters. **1992**, *65* p. 51-54.
69. Zhu, J.;Jiang, Y.;Yang, X.;Wang, S.;Xie, C.;Li, X.;Li, Y.;Chen, Y.;Wang, X.;Meng, Y.;Zhu, M.;Wu, R.;Huang, C.;Ma, X.;Geng, S.;Wu, J., Zhong, C. Wnt/β-catenin pathway mediates (−)-Epigallocatechin-3-gallate (EGCG) inhibition of lung cancer stem cells. Biochemical and Biophysical Research Communications. **2017**, *482* p. 15-21.
70. Oya, Y.;Mondal, A.;Rawangkan, A.;Umsumarng, S.;Iida, K.;Watanabe, T.;Kanno, M.;Suzuki, K.;Li, Z.;Kagechika, H.;Shudo, K.;Fujiki, H., Suganuma, M. Down-regulation of histone deacetylase 4, −5 and −6 as a mechanism of synergistic enhancement of apoptosis in human lung cancer cells treated with the combination of a synthetic retinoid, Am80 and green tea catechin. The Journal of Nutritional Biochemistry. **2017**, *42* p. 7-16.
71. Sazuka, M.;Murakami, S.;Isemura, M.;Satoh, K., Nukiwa, T. Inhibitory effects of green tea infusion on in vitro invasion and in vivo metastasis of mouse lung carcinoma cells. Cancer Letters. **1995**, *98* p. 27-31.
72. Leanderson, P.;Faresjö, Å.O., Tagesson, C. Green tea polyphenols inhibit oxidant-induced DNA strand breakage in cultured lung cells. Free Radical Biology and Medicine. **1997**, *23* p. 235-242.

73. Suganuma, M.;Okabe, S.;Sueoka, N.;Sueoka, E.;Matsuyama, S.;Imai, K.;Nakachi, K., Fujiki, H. Green tea and cancer chemoprevention. Mutation Research/Fundamental and Molecular Mechanisms of Mutagenesis. **1999**, *428* p. 339-344.
74. Huang, S.-F.;Horng, C.-T.;Hsieh, Y.-S.;Hsieh, Y.-H.;Chu, S.-C., Chen, P.-N. Epicatechin-3-gallate reverses TGF-β1-induced epithelial-to-mesenchymal transition and inhibits cell invasion and protease activities in human lung cancer cells. Food and Chemical Toxicology. **2016**, *94* p. 1-10.
75. Fujie, K.;Aoki, T.;Ito, Y., Maeda, S. Sister-chromatid exchanges induced by trihalomethanes in rat erythroblastic cells and their suppression by crude catechin extracted from green tea. Mutation Research/Genetic Toxicology. **1993**, *300* p. 241-246.
76. Otsuka, T.;Ogo, T.;Eto, T.;Asano, Y.;Suganuma, M., Niho, Y. Growth inhibition of leukemic cells by (−)-epigallocatechin gallate, the main constituent of green tea. Life Sciences. **1998**, *63* p. 1397-1403.
77. Lung, H.L.;Ip, W.K.;Wong, C.K.;Mak, N.K.;Chen, Z.Y., Leung, K.N. Anti-proliferative and differentiation-inducing activities of the green tea catechin epigallocatechin-3-gallate (EGCG) on the human eosinophilic leukemia EoL-1 cell line. Life Sciences. **2002**, *72* p. 257-268.
78. Cornwall, S.;Cull, G.;Joske, D., Ghassemifar, R. Green tea polyphenol "epigallocatechin-3-gallate", differentially induces apoptosis in CLL B-and T-Cells but not in healthy B-and T-Cells in a dose dependant manner. Leukemia Research. **2016**, *51* p. 56-61.
79. Parodi, S.;Merlo, D.F., Stagnaro, E. Coffee and tea consumption and risk of leukaemia in an adult population: A reanalysis of the Italian multicentre case-control study. Cancer Epidemiology. **2017**, *47* p. 81-87.
80. Hirose, M.;Hoshiya, T.;Akagi, K.;Futakuchi, M., Ito, N. Inhibition of mammary gland carcinogenesis by green tea catechins and other naturally occurring antioxidants in female Sprague-Dawley rats pretreated with 7, 12-dimethylbenz [a] anthracene. Cancer Letters. **1994**, *83* p. 149-156.
81. Hirose, M.;Mizoguchi, Y.;Yaono, M.;Tanaka, H.;Yamaguchi, T., Shirai, T. Effects of green tea catechins on the progression or late promotion stage of mammary gland carcinogenesis in female Sprague-Dawley rats pretreated with 7, 12-dimethylbenz (a) anthracene. Cancer Letters. **1997**, *112* p. 141-147.
82. Tanaka, H.;Hirose, M.;Kawabe, M.;Sano, M.;Takesada, Y.;Hagiwara, A., Shirai, T. Post-initiation inhibitory effects of green tea catechins on 7, 12-dimethylbenz [a] anthracene-induced mammary gland carcinogenesis in female Sprague–Dawley rats. Cancer Letters. **1997**, *116* p. 47-52.
83. Inoue, M.;Tajima, K.;Mizutani, M.;Iwata, H.;Iwase, T.;Miura, S.;Hirose, K.;Hamajima, N., Tominaga, S. Regular consumption of green tea and the risk of breast cancer recurrence: follow-up study from the Hospital-based Epidemiologic Research Program at Aichi Cancer Center (HERPACC), Japan. Cancer Letters. **2001**, *167* p. 175-182.

84. Zand, R.S.R.;Jenkins, D.J.;Brown, T.J., Diamandis, E.P. Flavonoids can block PSA production by breast and prostate cancer cell lines. Clinica Chimica Acta. **2002**, *317* p. 17-26.
85. Kao, G.;Kodra, A.;Kim, M., Solin, L. 2054: Effects of Green Tea Extract on Protein and mRNA Expression in Human Breast Cancer Cells: Impairment of the DNA Damage Response? International Journal of Radiation Oncology* Biology* Physics. **2006**, *66* p. S240.
86. Liberto, M.,Cobrinik, D. Growth factor-dependent induction of p21 CIP1 by the green tea polyphenol, epigallocatechin gallate. Cancer Letters. **2000**, *154* p. 151-161.
87. Abd El-Rahman, S.S.;Shehab, G., Nashaat, H. Epigallocatechin-3-Gallate: The Prospective Targeting of Cscs and Preventing Metastasis of Chemically-Induced Mammary Cancer in Rats☆. The American Journal of the Medical Sciences.
88. Li, W.;He, N.;Tian, L.;Shi, X., Yang, X. Inhibitory effects of polyphenol-enriched extract from Ziyang tea against human breast cancer MCF-7 cells through reactive oxygen species-dependent mitochondria molecular mechanism. Journal of Food and Drug Analysis. **2016**, *24* p. 527-538.
89. Yang, F.;Oz, H.S.;Barve, S.;De Villiers, W.J.;McClain, C.J., Varilek, G.W. The green tea polyphenol (−)-epigallocatechin-3-gallate blocks nuclear factor-κB activation by inhibiting IκB kinase activity in the intestinal epithelial cell line IEC-6. Molecular Pharmacology. **2001**, *60* p. 528-533.
90. Klaunig, J.E. Chemopreventive effects of green tea components on hepatic carcinogenesis. Preventive Medicine. **1992**, *21* p. 510-519.
91. Hasegawa, R.;Chujo, T.;Sai-Kato, K.;Umemura, T.;Tanimura, A., Kurokawa, Y. Preventive effects of green tea against liver oxidative DNA damage and hepatotoxicity in rats treated with 2-nitropropane. Food and Chemical Toxicology. **1995**, *33* p. 961-970.
92. Srivastava, R.;Husain, M.;Hasan, S., Athar, M. Green tea polyphenols and tannic acid act as potent inhibitors of phorbol ester-induced nitric oxide generation in rat hepatocytes independent of their antioxidant properties. Cancer Letters. **2000**, *153* p. 1-5.
93. Sadzuka, Y.;Sugiyama, T.;Miyagishima, A.;Nozawa, Y., Hirota, S. The effects of theanine, as a novel biochemical modulator, on the antitumor activity of adriamycin. Cancer Letters. **1996**, *105* p. 203-209.
94. Kennedy, D.O.;Nishimura, S.;Hasuma, T.;Yano, Y.;Otani, S., Matsui-Yuasa, I. Involvement of protein tyrosine phosphorylation in the effect of green tea polyphenols on Ehrlich ascites tumor cells in vitro. Chemico-Biological Interactions. **1998**, *110* p. 159-172.
95. Kennedy, D.O.;Kojima, A.;Yano, Y.;Hasuma, T.;Otani, S., Matsui-Yuasa, I. Growth inhibitory effect of green tea extract in Ehrlich ascites tumor cells involves cytochrome c release and caspase activation. Cancer letters. **2001**, *166* p. 9-15.

96. Paschka, A.G.;Butler, R., Young, C.Y.-F. Induction of apoptosis in prostate cancer cell lines by the green tea component,(−)-epigallocatechin-3-gallate. Cancer Letters. **1998**, *130* p. 1-7.
97. Gupta, S.;Ahmad, N.;Nieminen, A.-L., Mukhtar, H. Growth inhibition, cell-cycle dysregulation, and induction of apoptosis by green tea constituent (-)-epigallocatechin-3-gallate in androgen-sensitive and androgen-insensitive human prostate carcinoma cells. Toxicology and Applied Pharmacology. **2000**, *164* p. 82-90.
98. Wang, S.I.,Mukhtar, H. Gene expression profile in human prostate LNCaP cancer cells by (−) epigallocatechin-3-gallate. Cancer Letters. **2002**, *182* p. 43-51.
99. Ohishi, T.;Kishimoto, Y.;Miura, N.;Shiota, G.;Kohri, T.;Hara, Y.;Hasegawa, J., Isemura, M. Synergistic effects of (-)-epigallocatechin gallate with sulindac against colon carcinogenesis of rats treated with azoxymethane. Cancer Letters. **2002**, *177* p. 49-56.
100. Gupta, S.;Ahmad, N., Mukhtar, H. *Prostate cancer chemoprevention by green tea*. in *Seminars in urologic oncology*. 1999. W.B. Saunders Company.
101. Chung, L.;Cheung, T.;Kong, S.;Fung, K.;Choy, Y.;Chan, Z., Kwok, T. Induction of apoptosis by green tea catechins in human prostate cancer DU145 cells. Life Sciences. **2001**, *68* p. 1207-1214.
102. Sawada, N. Risk and preventive factors for prostate cancer in Japan: The Japan Public Health Center-based prospective (JPHC) study. Journal of Epidemiology. **2017**, *27* p. 2-7.
103. Sugiyama, T.,Sadzuka, Y. Enhancing effects of green tea components on the antitumor activity of adriamycin against M5076 ovarian sarcoma. Cancer Letters. **1998**, *133* p. 19-26.
104. Leung, A.C.Y.;Cook, L.S.;Swenerton, K.;Gilks, B.;Gallagher, R.P.;Magliocco, A.;Steed, H.;Köbel, M.;Nation, J.;Brooks-Wilson, A., Le, N.D. Tea, coffee, and caffeinated beverage consumption and risk of epithelial ovarian cancers. Cancer Epidemiology. **2016**, *45* p. 119-125.
105. Qin, J.;Xie, L.-P.;Zheng, X.-Y.;Wang, Y.-B.;Bai, Y.;Shen, H.-F.;Li, L.-C., Dahiya, R. A component of green tea, (−)-epigallocatechin-3-gallate, promotes apoptosis in T24 human bladder cancer cells via modulation of the PI3K/Akt pathway and Bcl-2 family proteins. Biochemical and Biophysical Research Communications. **2007**, *354* p. 852-857.
106. Luo, K.-W.;Wei, C.;Lung, W.-Y.;Wei, X.-Y.;Cheng, B.-H.;Cai, Z.-M., Huang, W.-R. EGCG inhibited bladder cancer SW780 cell proliferation and migration both in vitro and in vivo via down-regulation of NF-κB and MMP-9. The Journal of Nutritional Biochemistry. **2017**, *41* p. 56-64.
107. Sachinidis, A.;Seul, C.;Seewald, S.;Ahn, H.-Y.;Ko, Y., Vetter, H. Green tea compounds inhibit tyrosine phosphorylation of PDGF β-receptor and transformation of A172 human glioblastoma. FEBS Letters. **2000**, *471* p. 51-55.

108. Annabi, B.;Lachambre, M.-P.;Bousquet-Gagnon, N.;Pagé, M.;Gingras, D., Béliveau, R. Green tea polyphenol (−)-epigallocatechin 3-gallate inhibits MMP-2 secretion and MT1-MMP-driven migration in glioblastoma cells. Biochimica et Biophysica Acta (BBA)-Molecular Cell Research. **2002**, *1542* p. 209-220.
109. Mazzio, E.A.;Bauer, D.;Mendonca, P.;Taka, E., Soliman, K.F.A. Natural product HTP screening for attenuation of cytokine-induced neutrophil chemo attractants (CINCs) and NO2 − in LPS/IFNγ activated glioma cells. Journal of Neuroimmunology. **2017**, *302* p. 10-19.
110. Dashwood, W.-M.;Orner, G.A., Dashwood, R.H. Inhibition of β-catenin/Tcf activity by white tea, green tea, and epigallocatechin-3-gallate (EGCG): minor contribution of H 2 O 2 at physiologically relevant EGCG concentrations. Biochemical and Biophysical Research Communications. **2002**, *296* p. 584-588.
111. Carvalho, M.;Jerónimo, C.;Valentão, P.;Andrade, P.B., Silva, B.M. Green tea: A promising anticancer agent for renal cell carcinoma. Food Chemistry. **2010**, *122* p. 49-54.
112. Garcia, F.;Feugang, J.M.;Wang, J.;Cheng, S.J., Zou, C.P. Apoptosis induction by green tea compounds in cervical cancer cells. European Journal of Cancer Supplements. **2006**, *4* p. 58.
113. Shin, Y.S.;Kang, S.U.;Park, J.K.;Kim, Y.E.;Kim, Y.S.;Baek, S.J.;Lee, S.-H., Kim, C.-H. Anti-cancer effect of (-)-epigallocatechin-3-gallate (EGCG) in head and neck cancer through repression of transactivation and enhanced degradation of β-catenin. Phytomedicine. **2016**, *23* p. 1344-1355.
114. Yap, J.;Luesley, D.;Woodman, C., Dawson, C. The effectiveness of epigallocathechin-3-gallate for treatment of human papillomavirus-driven epithelial neoplasms: a preclinical study. The Lancet. **2017**, *389, Supplement 1* p. S102.
115. Ikigai, H.;Nakae, T.;Hara, Y., Shimamura, T. Bactericidal catechins damage the lipid bilayer. Biochimica et Biophysica Acta (BBA)-Biomembranes. **1993**, *1147* p. 132-136.
116. Anand, J.,Rai, N. Anticandidal synergistic activity of green tea catechins, antimycotics and copper sulphate as a mean of combinational drug therapy against candidiasis. Journal of Medical Mycology. **2017**, *27* p. 33-45.
117. Sugita-Konishi, Y.;Hara-Kudo, Y.;Amano, F.;Okubo, T.;Aoi, N.;Iwaki, M., Kumagai, S. Epigallocatechin gallate and gallocatechin gallate in green tea catechins inhibit extracellular release of Vero toxin from enterohemorrhagic Escherichia coli O157: H7. Biochimica et Biophysica Acta (BBA)-General Subjects. **1999**, *1472* p. 42-50.
118. Tsou, L.K.;Yount, J.S., Hang, H.C. Epigallocatechin-3-gallate inhibits bacterial virulence and invasion of host cells. Bioorganic & Medicinal Chemistry. **2017**,

119. Dyer, P.D.R.;Kotha, A.K.;Gollings, A.S.;Shorter, S.A.;Shepherd, T.R.;Pettit, M.W.;Alexander, B.D.;Getti, G.T.M.;El-Daher, S.;Baillie, L., Richardson, S.C.W. An in vitro evaluation of epigallocatechin gallate (eGCG) as a biocompatible inhibitor of ricin toxin. Biochimica et Biophysica Acta (BBA)-General Subjects. **2016**, *1860* p. 1541-1550.
120. Carneiro, B.M.;Batista, M.N.;Braga, A.C.S.;Nogueira, M.L., Rahal, P. The green tea molecule EGCG inhibits Zika virus entry. Virology. **2016**, *496* p. 215-218.
121. Lee, S.;Razqan, G.S.A., Kwon, D.H. Antibacterial activity of epigallocatechin-3-gallate (EGCG) and its synergism with β-lactam antibiotics sensitizing carbapenem-associated multidrug resistant clinical isolates of Acinetobacter baumannii. Phytomedicine. **2017**, *24* p. 49-55.
122. Nibir, Y.M.;Sumit, A.F.;Akhand, A.A.;Ahsan, N., Hossain, M.S. Comparative assessment of total polyphenols, antioxidant and antimicrobial activity of different tea varieties of Bangladesh. Asian Pacific Journal of Tropical Biomedicine. **2017**, *7* p. 352-357.
123. Sakanaka, S.;Juneja, L.R., Taniguchi, M. Antimicrobial effects of green tea polyphenols on thermophilic spore-forming bacteria. Journal of Bioscience and Bioengineering. **2000**, *90* p. 81-85.
124. Piva, G.;Fracassetti, D.;Tirelli, A.;Mascheroni, E.;Musatti, A.;Inglese, P.;Piergiovanni, L., Rollini, M. Evaluation of the antioxidant/antimicrobial performance of Posidonia oceanica in comparison with three commercial natural extracts and as a treatment on fresh-cut peaches (Prunus persica Batsch). Postharvest Biology and Technology. **2017**, *124* p. 54-61.
125. Rasheed, A.,Haider, M. Antibacterial activity of *Camellia sinensis* extracts against dental caries. Archives of Pharmacal Research. **1998**, *21* p. 348-352.
126. Horiba, N.;Maekawa, Y.;Ito, M.;Matsumoto, T., Nakamura, H. A pilot study of Japanese green tea as a medicament: antibacterial and bactericidal effects. Journal of Endodontics. **1991**, *17* p. 122-124.
127. Hui, X.;Liu, H.;Tian, F.-L.;Li, F.-F.;Li, H., Gao, W.-Y. Inhibition of green tea and the catechins against 1-deoxy-d-xylulose 5-phosphate reductoisomerase, the key enzyme of the MEP terpenoid biosynthetic pathway. Fitoterapia. **2016**, *113* p. 80-84.
128. Yun, J.,Lee, D.G. Role of potassium channels in chlorogenic acid-induced apoptotic volume decrease and cell cycle arrest in Candida albicans. Biochimica et Biophysica Acta (BBA)-General Subjects. **2017**, *1861* p. 585-592.
129. Ferraris, S.;Miola, M.;Cochis, A.;Azzimonti, B.;Rimondini, L.;Prenesti, E., Vernè, E. In situ reduction of antibacterial silver ions to metallic silver nanoparticles on bioactive glasses functionalized with polyphenols. Applied Surface Science. **2017**, *396* p. 461-470.

130. Hartjen, P.;Frerk, S.;Hauber, I.;Matzat, V.;Thomssen, A.;Holstermann, B.;Hohenberg, H.;Schulze, W.;zur Wiesch, J.S., van Lunzen, J. Assessment of the range of the HIV-1 infectivity enhancing effect of individual human semen specimen and the range of inhibition by EGCG. AIDS Research and Therapy. **2012**, *9* p. 2.
131. Nance, C.L.;Yeh, R.;Paul, M.;Seeborg, F.O.;Giordano, T.;Arduino, R.;Crutchley, R.;Mata, M.;Leechawengwongs, E., Shearer, W.T. Translational Medicine in HIV-1 Infection: Preclinical and Clinical Development of the Green Tea Catechin, Epigallocatechin Gallate, as Therapy and Immunological Signatures. Journal of Allergy and Clinical Immunology. **2017**, *139* p. AB209.
132. Bin Dajem, S.M.;Shati, A.A.;Adly, M.A.;Ahmed, O.M.;Ibrahim, E.H., Mostafa, O.M.S. Green tea (Camellia sinesis) ameliorates female Schistosoma mansoni-induced changes in the liver of Balb/C mice. Saudi Journal of Biological Sciences. **2011**, *18* p. 361-368.
133. Wickramasinghe, S.;Yatawara, L.;Nagataki, M., Agatsuma, T. Arginine kinase in Toxocara canis: Exon–intron organization, functional analysis of site-directed mutants and evaluation of putative enzyme inhibitors. Asian Pacific Journal of Tropical Medicine. **2016**, *9* p. 995-1001.
134. Vinson, J.A.,Dabbagh, Y.A. Effect of green and black tea supplementation on lipids, lipid oxidation and fibrinogen in the hamster: mechanisms for the epidemiological benefits of tea drinking. FEBS Letters. **1998**, *433* p. 44-46.
135. Yang, T.,Koo, M. Inhibitory effect of Chinese green tea on endothelial cell-induced LDL oxidation. Atherosclerosis. **2000**, *148* p. 67-73.
136. Tsutsui, Y.;Katanasaka, Y.;Sunagawa, Y.;Inoue, T.;Moriwaki, H.;Shimizu, Y.;Narushima, S.;Maeda, T.;Wada, H.;Hasegawa, K., Morimoto, T. GW27-e0925 KOSEN-CHA, a functional green tea, improves obesity and vascular function. Journal of the American College of Cardiology. **2016**, *68* p. C164-C165.
137. Imbe, H.;Sano, H.;Miyawaki, M.;Fujisawa, R.;Miyasato, M.;Nakatsuji, F.;Haseda, F.;Tanimoto, K.;Terasaki, J.;Maeda-Yamamoto, M.;Tachibana, H., Hanafusa, T. "Benifuuki" green tea, containing O-methylated EGCG, reduces serum low-density lipoprotein cholesterol and lectin-like oxidized low-density lipoprotein receptor-1 ligands containing apolipoprotein B: A double-blind, placebo-controlled randomized trial. Journal of Functional Foods. **2016**, *25* p. 25-37.
138. Grimaldi, V.;De Pascale, M.R.;Zullo, A.;Soricelli, A.;Infante, T.;Mancini, F.P., Napoli, C. Evidence of epigenetic tags in cardiac fibrosis. Journal of Cardiology. **2017**, *69* p. 401-408.
139. Miura, Y.;Chiba, T.;Miura, S.;Tomita, I.;Umegaki, K.;Ikeda, M., Tomita, T. Green tea polyphenols (flavan 3-ols) prevent oxidative modification of low density lipoproteins: an ex vivo study in humans. The Journal of Nutritional Biochemistry. **2000**, *11* p. 216-222.

140. Kokubo, Y.;Iso, H.;Saito, I.;Yamagishi, K.;Yatsuya, H.;Ishihara, J.;Inoue, M., Tsugane, S. The impact of green tea and coffee consumption on the reduced risk of stroke incidence in Japanese population. Stroke. **2013**, *44* p. 1369-1374.
141. Sasazuki, S.;Kodama, H.;Yoshimasu, K.;Liu, Y.;Washio, M.;Tanaka, K.;Tokunaga, S.;Kono, S.;Arai, H., Doi, Y. Relation between green tea consumption and the severity of coronary atherosclerosis among Japanese men and women. Annals of Epidemiology. **2000**, *10* p. 401-408.
142. Hirano, R.;Momiyama, Y.;Takahashi, R.;Taniguchi, H.;Kondo, K.;Nakamura, H., Ohsuzu, F. Comparison of green tea intake in Japanese patients with and without angiographic coronary artery disease. The American Journal of Cardiology. **2002**, *90* p. 1150-1153.
143. Wang, Z.-M.;Zhou, B.;Wang, Y.-S.;Gong, Q.-Y.;Wang, Q.-M.;Yan, J.-J.;Gao, W., Wang, L.-S. Black and green tea consumption and the risk of coronary artery disease: a meta-analysis. The American Journal of Clinical Nutrition. **2011**, *93* p. 506-515.
144. Lustosa, B.B.;Polegato, B.;Minicucci, M.;Rafacho, B.;Santos, P.P.;Fernandes, A.A.;Okoshi, K.;Batista, D.;Modesto, P.;Gonçalves, A.;Pereira, E.J.;Pires, V.;Paiva, S.;Zornoff, L., Azevedo, P.S. Green tea (Cammellia sinensis) attenuates ventricular remodeling after experimental myocardial infarction. International Journal of Cardiology. **2016**, *225* p. 147-153.
145. Bordoni, A.;Hrelia, S.;Angeloni, C.;Giordano, E.;Guarnieri, C.;Caldarera, C.M., Biagi, P.L. Green tea protection of hypoxia/reoxygenation injury in cultured cardiac cells. The Journal of Nutritional Biochemistry. **2002**, *13* p. 103-111.
146. Cheng, T.O. All teas are not created equal: The Chinese green tea and cardiovascular health. International Journal of Cardiology. **2006**, *108* p. 301-308.
147. Tomé-Carneiro, J.,Visioli, F. Polyphenol-based nutraceuticals for the prevention and treatment of cardiovascular disease: Review of human evidence. Phytomedicine. **2016**, *23* p. 1145-1174.
148. Hrelia, S.;Bordoni, A.;Angeloni, C.;Leoncini, E.;Toschi, T.G.;Lercker, G., Biagi, P. Green tea extracts can counteract the modification of fatty acid composition induced by doxorubicin in cultured cardiomyocytes. Prostaglandins, Leukotrienes and Essential Fatty Acids. **2002**, *66* p. 519-524.
149. Muramatsu, K.;Fukuyo, M., Hara, Y. Effect of green tea catechins on plasma cholesterol level in cholesterol-fed rats. Journal of Nutritional Science and Vitaminology. **1986**, *32* p. 613-622.
150. Antonello, M.;Montemurro, D.;Bolognesi, M.;Di Pascoli, M.;Piva, A.;Grego, F.;Sticchi, D.;Giuliani, L.;Garbisa, S., Rossi, G.P. Prevention of Hypertension, Cardiovascular Damage and Endothelial Dysfunction with Green Tea Extracts. American Journal of Hypertension. **2007**, *20* p. 1321-1328.

151. Yao, Y.-F.;Liu, X.;Li, W.-J.;Shi, Z.-W.;Yan, Y.-X.;Wang, L.-F.;Chen, M., Xie, M.-Y. (−)-Epigallocatechin-3-gallate alleviates doxorubicin-induced cardiotoxicity in sarcoma 180 tumor-bearing mice. Life Sciences. **2016**,
152. Sun, T.-L.;Liu, Z.;Qi, Z.-J.;Huang, Y.-P.;Gao, X.-Q., Zhang, Y.-Y. (-)-Epigallocatechin-3-gallate (EGCG) attenuates arsenic-induced cardiotoxicity in rats. Food and Chemical Toxicology. **2016**, *93* p. 102-110.
153. Oyama, J.-i.;Shiraki, A.;Nishikido, T.;Maeda, T.;Komoda, H.;Shimizu, T.;Makino, N., Node, K. EGCG, a green tea catechin, attenuates the progression of heart failure induced by the heart/muscle-specific deletion of MnSOD in mice. Journal of Cardiology. **2017**, *69* p. 417-427.
154. Ale-Agha, N.;Stahl, W., Sies, H. (−)-Epicatechin effects in rat liver epithelial cells: stimulation of gap junctional communication and counteraction of its loss due to the tumor promoter 12-O-tetradecanoylphorbol-13-acetate. Biochemical Pharmacology. **2002**, *63* p. 2145-2149.
155. Dower, J.I.;Geleijnse, J.M.;Gijsbers, L.;Zock, P.L.;Kromhout, D., Hollman, P.C. Effects of the pure flavonoids epicatechin and quercetin on vascular function and cardiometabolic health: a randomized, double-blind, placebo-controlled, crossover trial. The American journal of clinical nutrition. **2015**, *101* p. 914-921.
156. Tijburg, L.B.;Wiseman, S.A.;Meijer, G.W., Weststrate, J.A. Effects of green tea, black tea and dietary lipophilic antioxidants on LDL oxidizability and atherosclerosis in hypercholesterolaemic rabbits. Atherosclerosis. **1997**, *135* p. 37-47.
157. Ali, M.;Afzal, M.;Gubler, C., Burka, J.F. A potent thromboxane formation inhibitor in green tea leaves. Prostaglandins, Leukotrienes and Essential Fatty Acids. **1990**, *40* p. 281-283.
158. Yokozawa, T.,Dong, E. Influence of green tea and its three major components upon low-density lipoprotein oxidation. Experimental and Toxicologic Pathology. **1997**, *49* p. 329-335.
159. Abe, I.;Seki, T.;Umehara, K.;Miyase, T.;Noguchi, H.;Sakakibara, J., Ono, T. Green tea polyphenols: novel and potent inhibitors of squalene epoxidase. Biochemical and Biophysical Research Communications. **2000**, *268* p. 767-771.
160. Liu, Z.-Q.;Ma, L.-P.;Zhou, B.;Yang, L., Liu, Z.-L. Antioxidative effects of green tea polyphenols on free radical initiated and photosensitized peroxidation of human low density lipoprotein. Chemistry and Physics of Lipids. **2000**, *106* p. 53-63.
161. Pang, J.;Zhang, Z.;Zheng, T.-z.;Bassig, B.A.;Mao, C.;Liu, X.;Zhu, Y.;Shi, K.;Ge, J., Yang, Y.-j. Green tea consumption and risk of cardiovascular and ischemic related diseases: a meta-analysis. International Journal of Cardiology. **2016**, *202* p. 967-974.
162. Kasaoka, S.;Hase, K.;Morita, T., Kiriyama, S. Green tea flavonoids inhibit the LDL oxidation in osteogenic disordered rats fed a marginal ascorbic acid in diet. The Journal of Nutritional Biochemistry. **2002**, *13* p. 96-102.

163. Yoshikawa, M.;Morikawa, T.;Yamamoto, K.;Kato, Y.;Nagatomo, A., Matsuda, H. Floratheasaponins A− C, Acylated Oleanane-Type Triterpene Oligoglycosides with Anti-hyperlipidemic Activities from Flowers of the Tea Plant (Camellia sinensis) 1. Journal of Natural Products. **2005**, *68* p. 1360-1365.
164. Abe, Y.;Umemura, S.;Sugimoto, K.-i.;Hirawa, N.;Kato, Y.;Yokoyama, N.;Yokoyama, T.;Iwai, J., Ishii, M. Effect of green tea rich in γ-aminobutyric acid on blood pressure of Dahl salt-sensitive rats. American Journal of Hypertension. **1995**, *8* p. 74-79.
165. Juneja, L.R.;Chu, D.-C.;Okubo, T.;Nagato, Y., Yokogoshi, H. L-theanine—a unique amino acid of green tea and its relaxation effect in humans. Trends in Food Science & Technology. **1999**, *10* p. 199-204.
166. Bertoldi, M.;Gonsalvi, M., Voltattorni, C.B. Green tea polyphenols: novel irreversible inhibitors of dopa decarboxylase. Biochemical and Biophysical Research Communications. **2001**, *284* p. 90-93.
167. Yi, Q.-Y.;Li, H.-B.;Qi, J.;Yu, X.-J.;Huo, C.-J.;Li, X.;Bai, J.;Gao, H.-L.;Kou, B.;Liu, K.-L.;Zhang, D.-D.;Chen, W.-S.;Cui, W.;Zhu, G.-Q.;Shi, X.-L., Kang, Y.-M. Chronic infusion of epigallocatechin-3-O-gallate into the hypothalamic paraventricular nucleus attenuates hypertension and sympathoexcitation by restoring neurotransmitters and cytokines. Toxicology Letters. **2016**, *262* p. 105-113.
168. Ihm, S.-H.;Jang, S.-W.;Kim, O.-R.;Chang, K.;Oak, M.-H.;Lee, J.-O.;Lim, D.-Y., Kim, J.-H. Decaffeinated green tea extract improves hypertension and insulin resistance in a rat model of metabolic syndrome. Atherosclerosis. **2012**, *224* p. 377-383.
169. Kang, W.-S.;Lim, I.-H.;Yuk, D.-Y.;Chung, K.-H.;Park, J.-B.;Yoo, H.-S., Yun, Y.-P. Antithrombotic activities of green tea catechins and (−)-epigallocatechin gallate. Thrombosis Research. **1999**, *96* p. 229-237.
170. Ohnishi, S.T.;Ohnishi, T., Ogunmola, G.B. Green tea extract and aged garlic extract inhibit anion transport and sickle cell dehydration in vitro. Blood Cells, Molecules, and Diseases. **2001**, *27* p. 148-157.
171. Sabu, M.;Smitha, K., Kuttan, R. Anti-diabetic activity of green tea polyphenols and their role in reducing oxidative stress in experimental diabetes. Journal of Ethnopharmacology. **2002**, *83* p. 109-116.
172. Zhang, P.W.;Tian, C.;Xu, F.Y.;Chen, Z.;Burnside, R.;Yi, W.J.;Xiang, S.Y.;Xie, X.;Wu, N.N.;Yang, H.;Zhao, N.N.;Ye, X.L., Ying, C.J. Green Tea Polyphenols Alleviate Autophagy Inhibition Induced by High Glucose in Endothelial Cells. Biomedical and Environmental Sciences. **2016**, *29* p. 524-528.
173. Levy, Y.;Narotzki, B., Reznick, A.Z. Green tea, weight loss and physical activity. Clinical Nutrition. **2017**, *36* p. 315.
174. Cao, Y.;Cao, R., Bråkenhielm, E. Antiangiogenic mechanisms of diet-derived polyphenols. The Journal of Nutritional Biochemistry. **2002**, *13* p. 380-390.

175. Sampath, C.;Rashid, M.R.;Sang, S., Ahmedna, M. Green tea epigallocatechin 3-gallate alleviates hyperglycemia and reduces advanced glycation end products via nrf2 pathway in mice with high fat diet-induced obesity. Biomedicine & Pharmacotherapy. **2017**, *87* p. 73-81.
176. Pournourmohammadi, S.;Grimaldi, M.;Stridh, M.H.;Lavallard, V.;Waagepetersen, H.S.;Wollheim, C.B., Maechler, P. Epigallocatechin-3-gallate (EGCG) activates AMPK through the inhibition of glutamate dehydrogenase in muscle and pancreatic ß-cells: A potential beneficial effect in the pre-diabetic state? The International Journal of Biochemistry & Cell Biology. **2017**,
177. Rupasinghe, H.P.V.;Sekhon-Loodu, S.;Mantso, T., Panayiotidis, M.I. Phytochemicals in regulating fatty acid β-oxidation: Potential underlying mechanisms and their involvement in obesity and weight loss. Pharmacology & Therapeutics. **2016**, *165* p. 153-163.
178. Wu, M.;Liu, D.;Zeng, R.;Xian, T.;Lu, Y.;Zeng, G.;Sun, Z.;Huang, B., Huang, Q. Epigallocatechin-3-gallate inhibits adipogenesis through down-regulation of PPARγ and FAS expression mediated by PI3K-AKT signaling in 3T3-L1 cells. European Journal of Pharmacology. **2017**, *795* p. 134-142.
179. Kapoor, M.P.;Sugita, M.;Fukuzawa, Y., Okubo, T. Physiological effects of epigallocatechin-3-gallate (EGCG) on energy expenditure for prospective fat oxidation in humans: A systematic review and meta-analysis. The Journal of Nutritional Biochemistry. **2017**, *43* p. 1-10.
180. Yoshikawa, M.;Wang, T.;Sugimoto, S.;Nakamura, S.;Nagatomo, A.;Matsuda, H., Harima, S. Functional saponins in tea flower (flower buds of Camellia sinensis): gastroprotective and hypoglycemic effects of floratheasaponins and qualitative and quantitative analysis using HPLC. Yakugaku zasshi: Journal of the Pharmaceutical Society of Japan. **2008**, *128* p. 141-151.
181. Han, Q.;Yu, Q.-y.;Shi, J.;Xiong, C.-y.;Ling, Z.-j., He, P.-m. Molecular characterization and hypoglycemic activity of a novel water-soluble polysaccharide from tea (Camellia sinensis) flower. Carbohydrate Polymers. **2011**, *86* p. 797-805.
182. Sun, L.;Warren, F.J.;Netzel, G., Gidley, M.J. 3 or 3′-Galloyl substitution plays an important role in association of catechins and theaflavins with porcine pancreatic α-amylase: The kinetics of inhibition of α-amylase by tea polyphenols. Journal of Functional Foods. **2016**, *26* p. 144-156.
183. Bin, W.;Porrata-Maury, C.;Weiguo, M.;Bing, X.;Alegret-Rodríguez, M.;Yujing, Q., Hernández-Triana, M. Short term effect of the MA-PI 2 macrobiotic diet in type 2 diabetic adults of Beijing, China. Conference: 16th Latin American Congress of Nutrition. **2012**,

184. Narotzki, B.;Reznick, A.Z.;Navot-Mintzer, D.;Dagan, B., Levy, Y. Green tea and vitamin E enhance exercise-induced benefits in body composition, glucose homeostasis, and antioxidant status in elderly men and women. Journal of the American College of Nutrition. **2013**, *32* p. 31-40.
185. Yousaf, S.;Butt, M.;Suleria, H., Iqbal, M. The role of green tea extract and powder in mitigating metabolic syndromes with special reference to hyperglycemia and hypercholesterolemia. Food & Function. **2014**, *5* p. 545-556.
186. Wang, Z.-Y.;Huang, M.-T.;Ferraro, T.;Wong, C.-Q.;Lou, Y.-R.;Reuhl, K.;Iatropoulos, M.;Yang, C.S., Conney, A.H. Inhibitory effect of green tea in the drinking water on tumorigenesis by ultraviolet light and 12-O-tetradecanoylphorbol-13-acetate in the skin of SKH-1 mice. Cancer Research. **1992**, *52* p. 1162-1170.
187. Chen, I.J.;Liu, C.-Y.;Chiu, J.-P., Hsu, C.-H. Therapeutic effect of high-dose green tea extract on weight reduction: A randomized, double-blind, placebo-controlled clinical trial. Clinical Nutrition. **2016**, *35* p. 592-599.
188. Han, L.;Kimura, Y.;Kawashima, M.;Takaku, T.;Taniyama, T.;Hayashi, T.;Zheng, Y., Okuda, H. Anti-obesity effects in rodents of dietary teasaponin, a lipase inhibitor. International Journal of Obesity. **2001**, *25* p. 1459.
189. Auvichayapat, P.;Prapochanung, M.;Tunkamnerdthai, O.;Sripanidkulchai, B.-o.;Auvichayapat, N.;Thinkhamrop, B.;Kunhasura, S.;Wongpratoom, S.;Sinawat, S., Hongprapas, P. Effectiveness of green tea on weight reduction in obese Thais: A randomized, controlled trial. Physiology & Behavior. **2008**, *93* p. 486-491.
190. Xu, Y.;Ho, C.-T.;Amin, S.G.;Han, C., Chung, F.-L. Inhibition of tobacco-specific nitrosamine-induced lung tumorigenesis in A/J mice by green tea and its major polyphenol as antioxidants. Cancer Research. **1992**, *52* p. 3875-3879.
191. Janssens, P.L.H.R.;Hursel, R., Westerterp-Plantenga, M.S. Nutraceuticals for body-weight management: The role of green tea catechins. Physiology & Behavior. **2016**, *162* p. 83-87.
192. Afzalpour, M.E.;Ghasemi, E., Zarban, A. Effects of 10 weeks of high intensity interval training and green tea supplementation on serum levels of Sirtuin-1 and peroxisome proliferator-activated receptor gamma co-activator 1-alpha in overweight women. Science & Sports. **2017**,
193. Huang, Y.;Chan, N.W.K.;Lau, C.W.;Yao, X.Q.;Chan, F.L., Chen, Z.Y. Involvement of endothelium/nitric oxide in vasorelaxation induced by purified green tea (−) epicatechin. Biochimica et Biophysica Acta (BBA)-General Subjects. **1999**, *1427* p. 322-328.
194. Setozaki, S.;Minakata, K.;Masumoto, H.;Hirao, S.;Yamazaki, K.;Kuwahara, K.;Ikeda, T., Sakata, R. Prevention of abdominal aortic aneurysm progression by oral administration of green tea polyphenol in a rat model. Journal of Vascular Surgery. **2016**,
195. Shibata, K.;Moriyama, M.;Fukushima, T.;Kaetsu, A.;Miyazaki, M., Une, H. Green tea consumption and chronic atrophic gastritis: a cross-sectional study in a green tea production village. Journal of Epidemiology. **2000**, *10* p. 310-316.

196. Ahn, Y.-J.;Kawamura, T.;Kim, M.;Yamamoto, T., Mitsuoka, T. Tea polyphenols: selective growth inhibitors of Clostridium spp. Agricultural and Biological Chemistry. **1991**, *55* p. 1425-1426.
197. Jung, E.S.;Park, H.M.;Hyun, S.M.;Shon, J.-C.;Liu, K.-H.;Hwang, J.S., Lee, C.H. Modulation of gut microbiome by green tea diet and its correlation with metabolic changes. Drug Metabolism and Pharmacokinetics. **2017**, *32* p. S62.
198. Guo, Q.;Zhao, B.;Li, M.;Shen, S., Xin, W. Studies on protective mechanisms of four components of green tea polyphenols against lipid peroxidation in synaptosomes. Biochimica et Biophysica Acta (BBA)-Lipids and Lipid Metabolism. **1996**, *1304* p. 210-222.
199. Zhao, X.;Liu, F.;Jin, H.;Li, R.;Wang, Y.;Zhang, W.;Wang, H., Chen, W. Involvement of PKCα and ERK1/2 signaling pathways in EGCG's protection against stress-induced neural injuries in Wistar rats. Neuroscience. **2017**, *346* p. 226-237.
200. Lee, S.-R.;Suh, S.-I., Kim, S.-P. Protective effects of the green tea polyphenol (−)-epigallocatechin gallate against hippocampal neuronal damage after transient global ischemia in gerbils. Neuroscience Letters. **2000**, *287* p. 191-194.
201. Nagai, K.;Jiang, M.H.;Hada, J.;Nagata, T.;Yajima, Y.;Yamamoto, S., Nishizaki, T. (−)-Epigallocatechin gallate protects against NO stress-induced neuronal damage after ischemia by acting as an anti-oxidant. Brain Research. **2002**, *956* p. 319-322.
202. Bai, Q.;Lyu, Z.;Yang, X.;Pan, Z.;Lou, J., Dong, T. Epigallocatechin-3-gallate promotes angiogenesis via up-regulation of Nfr2 signaling pathway in a mouse model of ischemic stroke. Behavioural Brain Research. **2017**, *321* p. 79-86.
203. McConnell, J.S.;McConnell, R., Hossner, L. *Ultraviolet spectra of acetic acid, glycine, and glyphosate*. in *Proceedings Arkansas Academy of Science*. 1993.
204. Choi, J.-Y.;Park, C.-S.;Kim, D.-J.;Cho, M.-H.;Jin, B.-K.;Pie, J.-E., Chung, W.-G. Prevention of nitric oxide-mediated 1-methyl-4-phenyl-1, 2, 3, 6-tetrahydropyridine-induced Parkinson's disease in mice by tea phenolic epigallocatechin 3-gallate. NeuroToxicology. **2002**, *23* p. 367-374.
205. Choi, Y.-T.;Jung, C.-H.;Lee, S.-R.;Bae, J.-H.;Baek, W.-K.;Suh, M.-H.;Park, J.;Park, C.-W., Suh, S.-I. The green tea polyphenol (−)-epigallocatechin gallate attenuates β-amyloid-induced neurotoxicity in cultured hippocampal neurons. Life Sciences. **2001**, *70* p. 603-614.
206. Ahmed, I.;John, A.;Vijayasarathy, C.;Robin, M.A., Raza, H. Differential Modulation of Growth and Glutathione Metabolism in Cultured Rat Astrocytes by 4-Hydroxynonenal and Green Tea Polyphenol, Epigallocatechin-3-Gallate. NeuroToxicology. **2002**, *23* p. 289-300.
207. Gumay, A.R.;Bakri, S., Pudjonarko, D. The Effect of Green Tea Epigallocatechin-3-Gallate on Spatial Memory Function, Malondialdehyde and TNF-α Level in D-Galactose-Induced BALB/C Mice. Hiroshima Journal of Medical Sciences. **2018**, *67* p. 41-48.

208. Hong, J.T.;Ryu, S.R.;Kim, H.J.;Lee, J.K.;Lee, S.H.;Kim, D.B.;Yun, Y.P.;Ryu, J.H.;Lee, B.M., Kim, P.Y. Neuroprotective effect of green tea extract in experimental ischemia-reperfusion brain injury. Brain Research Bulletin. **2000**, *53* p. 743-749.
209. Hong, J.T.;Ryu, S.R.;Kim, H.J.;Lee, J.K.;Lee, S.H.;Yun, Y.P.;Lee, B.M., Kim, P.Y. Protective effect of green tea extract on ischemia/reperfusion-induced brain injury in Mongolian gerbils. Brain Research. **2001**, *888* p. 11-18.
210. Kaur, T.;Pathak, C.M.;Pandhi, P., Khanduja, K.L. Effects of green tea extract on learning, memory, behavior and acetylcholinesterase activity in young and old male rats. Brain and Cognition. **2008**, *67* p. 25-30.
211. Lavretsky, H. Lifestyle Medicine for Prevention of Cognitive Decline: Focus on Green Tea. The American Journal of Geriatric Psychiatry. **2016**, *24* p. 890-892.
212. Altermann, C.D.C.;Souza, M.A.;Schimidt, H.L.;Izaguirry, A.P.;Martins, A.;Garcia, A.;Santos, F.W., Mello-Carpes, P.B. Short-term green tea supplementation prevents recognition memory deficits and ameliorates hippocampal oxidative stress induced by different stroke models in rats. Brain Research Bulletin.
213. Arab, L.;Liu, W., Elashoff, D. Green and black tea consumption and risk of stroke. Stroke. **2009**, *40* p. 1786-1792.
214. Ben, P.;Zhang, Z.;Zhu, Y.;Xiong, A.;Gao, Y.;Mu, J.;Yin, Z., Luo, L. l-Theanine attenuates cadmium-induced neurotoxicity through the inhibition of oxidative damage and tau hyperphosphorylation. NeuroToxicology. **2016**, *57* p. 95-103.
215. Levites, Y.;Youdim, M.B.H.;Maor, G., Mandel, S. Attenuation of 6-hydroxydopamine (6-OHDA)-induced nuclear factor-kappaB (NF-κB) activation and cell death by tea extracts in neuronal cultures1. Biochemical Pharmacology. **2002**, *63* p. 21-29.
216. Weinreb, O.;Mandel, S.;Amit, T., Youdim, M.B. Neurological mechanisms of green tea polyphenols in Alzheimer's and Parkinson's diseases. The Journal of Nutritional Biochemistry. **2004**, *15* p. 506-516.
217. Unno, K.;Takabayashi, F.;Kishido, T., Oku, N. Suppressive effect of green tea catechins on morphologic and functional regression of the brain in aged mice with accelerated senescence (SAMP10). Experimental Gerontology. **2004**, *39* p. 1027-1034.
218. Jeon, S.-Y.;Bae, K.;Seong, Y.-H., Song, K.-S. Green tea catechins as a BACE1 (β-Secretase) inhibitor. Bioorganic & Medicinal Chemistry Letters. **2003**, *13* p. 3905-3908.
219. Geiser, R.J.;Chastain, S.E., Moss, M.A. Regulation of Bace1 Mrna Expression in Alzheimer'S Disease by Green Tea Catechins and Black Tea Theaflavins. Biophysical Journal. **2017**, *112* p. 362a.
220. Pervin, M.;Unno, K.;Nakagawa, A.;Takahashi, Y.;Iguchi, K.;Yamamoto, H.;Hoshino, M.;Hara, A.;Takagaki, A.;Nanjo, F.;Minami, A.;Imai, S., Nakamura, Y. Blood brain barrier permeability of (−)-epigallocatechin gallate, its proliferation-enhancing activity of human neuroblastoma SH-SY5Y cells, and its preventive effect on age-related cognitive dysfunction in mice. Biochemistry and Biophysics Reports. **2017**, *9* p. 180-186.

221. Suganuma, M.;Okabe, S.;Oniyama, M.;Tada, Y.;Ito, H., Fujiki, H. Wide distribution of [3H](-)-epigallocatechin gallate, a cancer preventive tea polyphenol, in mouse tissue. Carcinogenesis. **1998**, *19* p. 1771-1776.
222. Pan, C.-Y.;Kao, Y.-H., Fox, A.P. Enhancement of inward Ca 2+ currents in bovine chromaffin cells by green tea polyphenol extracts. Neurochemistry International. **2002**, *40* p. 131-137.
223. Unno, K.;Hara, A.;Nakagawa, A.;Iguchi, K.;Ohshio, M.;Morita, A., Nakamura, Y. Anti-stress effects of drinking green tea with lowered caffeine and enriched theanine, epigallocatechin and arginine on psychosocial stress induced adrenal hypertrophy in mice. Phytomedicine. **2016**, *23* p. 1365-1374.
224. Yang, F.;De Villiers, W.J.;McClain, C.J., Varilek, G.W. Green tea polyphenols block endotoxin-induced tumor necrosis factor-production and lethality in a murine model. The Journal of Nutrition. **1998**, *128* p. 2334-2340.
225. Sato, T.,Miyata, G. The nutraceutical benefit, part I: green tea. Nutrition. **2000**, *16* p. 315-317.
226. Chan, M.M.-Y.;Fong, D.;Ho, C.-T., Huang, H.-I. Inhibition of inducible nitric oxide synthase gene expression and enzyme activity by epigallocatechin gallate, a natural product from green tea. Biochemical pharmacology. **1997**, *54* p. 1281-1286.
227. Wang, Z.Y.;Khan, W.A.;Bickers, D.R., Mukhtar, H. Protection against polycyclic aromatic hydrocarbon-induced skin tumor initiation in mice by green tea polyphenols. Carcinogenesis. **1989**, *10* p. 411-415.
228. Smithson, J.;Kellick, K.A., Mergenhagen, K., *Chapter 16 - Nutritional Modulators of Pain in the Aging Population A2 - Watson, Ronald Ross*, in *Nutritional Modulators of Pain in the Aging Population*, S. Zibadi, Editor. 2017, Academic Press. p. 191-198.
229. Hashempur, M.H.;Sadrneshin, S.;Mosavat, S.H., Ashraf, A. Green tea (Camellia sinensis) for patients with knee osteoarthritis: A randomized open-label active-controlled clinical trial. Clinical Nutrition. **2016**,
230. Ashraf, A.;Sadrneshin, S.;Mosavat, S.H., Hashempur, M.H. Efficacy of the green tea (Camellia sinensis) tablet in knee osteoarthritis. European Journal of Integrative Medicine. **2016**, *8, Supplement 1* p. 10-11.
231. Maeda, Y. Inhibitory effects of tea extracts on histamine release from mast cells. Journal of the Food Hygienic Society of Japan. **1980**, *30* p. 295-299.
232. Akagi, M.;Fukuishi, N.;Tomoko, K.;SAGESAKA, Y.M., AKAGI, R. Anti-allergic effect of tea-leaf saponin (TLS) from tea leaves (Camellia sinensis var. sinensis). Biological and Pharmaceutical Bulletin. **1997**, *20* p. 565-567.
233. Yoshikawa, M.;Nakamura, S.;Kato, Y.;Matsuhira, K., Matsuda, H. Medicinal Flowers. XIV. 1) New acylated Oleanane-Type triterpene oligoglycosides with antiallergic activity from flower buds of chinese tea plant (Camellia sinensis). Chemical and Pharmaceutical Bulletin. **2007**, *55* p. 598-605.

234. Ng, H.L.H.;Premilovac, D.;Rattigan, S.;Richards, S.M.;Muniyappa, R.;Quon, M.J., Keske, M.A. Acute vascular and metabolic actions of the green tea polyphenol epigallocatechin 3-gallate in rat skeletal muscle. The Journal of Nutritional Biochemistry. **2017**, *40* p. 23-31.
235. Kim, A.R.;Kim, K.M.;Byun, M.R.;Hwang, J.-H.;Park, J.I.;Oh, H.T.;Jeong, M.G.;Hwang, E.S., Hong, J.-H. (-)-Epigallocatechin-3-gallate stimulates myogenic differentiation through TAZ activation. Biochemical and Biophysical Research Communications. **2017**,
236. Takahashi, H.;Suzuki, Y.;Mohamed, J.S.;Gotoh, T.;Pereira, S.L., Alway, S.E. Epigallocatechin-3-gallate increases autophagy signaling in resting and unloaded plantaris muscles but selectively suppresses autophagy protein abundance in reloaded muscles of aged rats. Experimental Gerontology. **2017**,
237. Nakagawa, H.;Wachi, M.;Woo, J.-T.;Kato, M.;Kasai, S.;Takahashi, F.;Lee, I.-S., Nagai, K. Fenton reaction is primarily involved in a mechanism of (−)-epigallocatechin-3-gallate to induce osteoclastic cell death. Biochemical and Biophysical Research Communications. **2002**, *292* p. 94-101.
238. Chu, C.;Deng, J.;Xiang, L.;Wu, Y.;Wei, X.;Qu, Y., Man, Y. Evaluation of epigallocatechin-3-gallate (EGCG) cross-linked collagen membranes and concerns on osteoblasts. Materials Science and Engineering: C. **2016**, *67* p. 386-394.
239. Kuroyanagi, G.;Tokuda, H.;Yamamoto, N.;Kainuma, S.;Fujita, K.;Ohguchi, R.;Kawabata, T.;Sakai, G.;Matsushima-Nishiwaki, R.;Harada, A.;Kozawa, O., Otsuka, T. (-)-Epigallocatechin gallate synergistically potentiates prostaglandin E2-stimulated osteoprotegerin synthesis in osteoblasts. Prostaglandins & Other Lipid Mediators. **2017**, *128–129* p. 27-33.
240. Ahmed, S.;Rahman, A.;Hasnain, A.;Lalonde, M.;Goldberg, V.M., Haqqi, T.M. Green tea polyphenol epigallocatechin-3-gallate inhibits the IL-1β-induced activity and expression of cyclooxygenase-2 and nitric oxide synthase-2 in human chondrocytes. Free Radical Biology and Medicine. **2002**, *33* p. 1097-1105.
